# Genome-Wide Characterization of Ascorbate Peroxidase Gene Family in Peanut (*Arachis hypogea* L.) Revealed Their Crucial Role in Growth and Multiple Stress Tolerance

**DOI:** 10.3389/fpls.2022.962182

**Published:** 2022-09-09

**Authors:** Ali Raza, Yasir Sharif, Kun Chen, Lihui Wang, Huiwen Fu, Yuhui Zhuang, Annapurna Chitikineni, Hua Chen, Chong Zhang, Rajeev K. Varshney, Weijian Zhuang

**Affiliations:** ^1^Key Laboratory of Ministry of Education for Genetics, Breeding and Multiple Utilization of Crops, Center of Legume Plant Genetics and Systems Biology, College of Agriculture, Oil Crops Research Institute, Fujian Agriculture and Forestry University (FAFU), Fuzhou, China; ^2^College of Plant Protection, Fujian Agriculture and Forestry University (FAFU), Fuzhou, China; ^3^College of Life Science, Fujian Agriculture and Forestry University (FAFU), Fuzhou, China; ^4^Murdoch’s Centre for Crop and Food Innovation, State Agricultural Biotechnology Centre, Food Futures Institute, Murdoch University, Murdoch, WA, Australia

**Keywords:** abiotic stress, antioxidant, drought, genomics, gene ontology, legume, miRNAs, stress responses

## Abstract

Ascorbate peroxidase (APX), an important antioxidant enzyme, plays a significant role in ROS scavenging by catalyzing the decrease of hydrogen peroxide under various environmental stresses. Nevertheless, information about the *APX* gene family and their evolutionary and functional attributes in peanut (*Arachis hypogea* L.) was not reported. Therefore, a comprehensive genome-wide study was performed to discover the *APX* genes in cultivated peanut genome. This study identified 166 *AhAPX* genes in the peanut genome, classified into 11 main groups. The gene duplication analysis showed that *AhAPX* genes had experienced segmental duplications and purifying selection pressure. Gene structure and motif investigation indicated that most of the *AhAPX* genes exhibited a comparatively well-preserved exon-intron pattern and motif configuration contained by the identical group. We discovered five phytohormones-, six abiotic stress-, and five growth and development-related *cis*-elements in the promoter regions of *AhAPX*. Fourteen putative ah-miRNAs from 12 families were identified, targeting 33 *AhAPX* genes. Furthermore, we identified 3,257 transcription factors from 38 families (including AP2, ARF, B3, bHLH, bZIP, ERF, MYB, NAC, WRKY, etc.) in 162 *AhAPX* genes. Gene ontology and KEGG enrichment analysis confirm the role of *AhAPX* genes in oxidoreductase activity, catalytic activity, cell junction, cellular response to stimulus and detoxification, biosynthesis of metabolites, and phenylpropanoid metabolism. Based on transcriptome datasets, some genes such as *AhAPX4/7/17/77/82/86/130/133* and *AhAPX160* showed significantly higher expression in diverse tissues/organs, i.e., flower, leaf, stem, roots, peg, testa, and cotyledon. Likewise, only a few genes, including *AhAPX4/17/19/55/59/82/101/102/137* and *AhAPX140*, were significantly upregulated under abiotic (drought and cold), and phytohormones (ethylene, abscisic acid, paclobutrazol, brassinolide, and salicylic acid) treatments. qRT-PCR-based expression profiling presented the parallel expression trends as generated from transcriptome datasets. Our discoveries gave new visions into the evolution of *APX* genes and provided a base for further functional examinations of the *AhAPX* genes in peanut breeding programs.

## Introduction

Plants are regularly subjected to various environmental factors (abiotic and biotic), which substantially influence crop productivity and cause challenges to food security (Sabagh et al., 2021; Mir et al., 2022; Raza et al., 2022a,b; Saeed et al., 2022; Sharma et al., 2022). These factors can enhance the generation of reactive oxygen species (ROS), damaging cellular systems and supermolecules consisting of DNA, proteins, and lipids, and ultimately leading to cell death (Fahad et al., 2015, 2017; Mittler, 2017; Hasanuzzaman et al., 2020). ROS are mainly produced in different locations including chloroplast, apoplast, plasma membrane, mitochondrion, endoplasmic reticulum, peroxisomes, and cell walls (Mittler, 2017; Hasanuzzaman et al., 2020). In plants, ROS are formed as chemical by-products due to the imperfect decline of oxygen metabolism. Further, ROS are considered as signaling elements that regulate stress tolerance mechanisms in plant molecular biology (Das and Roychoudhury, 2014; Mittler, 2017; Hasanuzzaman et al., 2020). Current progress has revealed that ROS homeostasis is essential for maintaining typical cellular characteristics (Mittler, 2017; Hasanuzzaman et al., 2020, 2021). Subsequently, for regular ROS signaling, plants have developed defense systems including enzymatic and non-enzymatic antioxidant enzymes to maintain the equilibrium between ROS-scavenging and production under stress conditions (Das and Roychoudhury, 2014; Mittler, 2017; Hasanuzzaman et al., 2020, 2021).

In plants, among diverse antioxidant enzymes entailed in ROS-scavenging mechanisms, ascorbate peroxidase (APX; EC, 1.11.1.11) belongs to the heme peroxidase superfamily (Hodges et al., 1999; Teixeira et al., 2004; Lazzarotto et al., 2011). In higher plants, APXs are one of the main antioxidant enzymes involved in regulating the ascorbate-glutathione cycle and take parts to scavenge hydrogen peroxide (H_2_O_2_) from chloroplast and the cytoplasm. Mainly, it utilized the ascorbic acid as an electron giver to scavenge H_2_O_2_ generated in plants and thus enhances tolerance to oxidative and other stresses in plants (Cao et al., 2017; Pandey et al., 2017; Hasanuzzaman et al., 2021; Raza et al., 2021a). Additionally, APX enzymes are automated by *APX* gene family involved in stress tolerance has been thoroughly explored in diverse plant species using various *in silico* approaches. For example, five *APX* genes have been discovered in wild watermelon (*Citrullus lanatus*) (Malambane et al., 2018); six in shrub (*Ammopiptanthus nanus*) (Wang et al., 2022); eight in rice (*Oryza sativa* L.) (Teixeira et al., 2004) and *Arabidopsis thaliana* (Panchuk et al., 2002, Panchuk et al., 2005); nine in sorghum (*Sorghum bicolor* L.) (Akbudak et al., 2018); 13 in kiwifruit (*Actinidia chinensis*) (Liao et al., 2020); 16 *APX* genes in tomato (*Solanum lycopersicum* L.) (Najami et al., 2008); 21 in wheat (*Triticum aestivum* L.) (Tyagi et al., 2020); and 26 in cotton (*Gossypium hirsutum* L.) (Tao et al., 2018). Nevertheless, the *APX* gene family in peanut (*Arachis hypogea* L.) has not been systematically reported, and their roles in peanut development and stress tolerance still remain ambiguous.

Cultivated peanut/groundnut (*A. hypogaea* L.), an allotetraploid crop, is one of the most valuable and economic oilseed food crops globally (Agarwal et al., 2018; Bertioli et al., 2019; Chen X. et al., 2019; Zhuang et al., 2019). This crop is being widely cultivated in the tropical and subtropical regions globally; however, several abiotic and biotic factors significantly affect its growth and production, including many important agronomic traits (Agarwal et al., 2018; Gangurde et al., 2020, 2021; Kumar et al., 2020; Pandey et al., 2020; Shasidhar et al., 2020; Sinha et al., 2020; Jadhav et al., 2021; Soni et al., 2021; Aravind et al., 2022; Bomireddy et al., 2022; Liu et al., 2022; Patel et al., 2022). Therefore, it is vital to identify new potential genes associated with multiple stress tolerance and trait improvement in peanut for better protein-rich food supply, particularly in Asian and African countries. In this regard, the recently sequenced peanut genome and recent advances in genomics-assisted breeding make it easier for us to carry out a comprehensive systematic analysis of new gene families (Varshney et al., 2019, 2020, 2021a,b). To our best knowledge, *APX* gene family was yet to be comprehensively characterized in peanut. Thus, the current study performed a genome-wide identification and characterized the *APX* gene family in peanut (*AhAPX*). Several *in silico* analysis, such as characterization, genomic evolution, gene structure, conserved motifs, *cis*-regulatory elements, putative miRNA and transcription factors, functional annotations, etc., were utilized to get insights into the novel roles of *AhAPX* genes. Furthermore, their expression profiling in diverse tissues/organs, under phytohormones and abiotic stress conditions were also performed using transcriptome and qRT-PCR techniques. In short, this report offered evolutionary and functional roles of *AhAPX* genes which could open new windows for further functional studies on the novel roles of *AhAPX* genes in peanut breeding programs under stress conditions.

## Materials and Methods

### Discovery and Physicochemical Features of *APX* Genes

As explained earlier (Li et al., 2021; Raza et al., 2021b; Su et al., 2021), two approaches, i.e., BLASTP and the Hidden Markov Model (HMM), were applied to identify *APX* genes in the peanut (*A. hypogea*) genome. The peanut genome sequence was taken from peanut Genome Resource (PGR) database^1^ (Zhuang et al., 2019). In the first approach, the sequences of eight *Arabidopsis thaliana APX* genes were gained from TAIR Arabidopsis genome database^2^ (Rhee et al., 2003). Then, these sequences were utilized as a query to perform the BLASTP against peanut genome. In the second approach, HMMER 3.1^3^ (Finn et al., 2015) software was employed to seek out the *APX* genes with default controls. Later, the HMM file of the ascorbic acid peroxidase domain (PF00141) was retrieved from the Pfam database^4^ (El-Gebali et al., 2019). Lastly, the sequences comprising the PF00141 domain were chosen as putative *APX* genes, and finally, 166 *AhAPX* genes were discovered by uniting the results obtained from both approaches in the peanut genome. Following the same approaches, *APX* genes were also discovered in diploid parents, i.e., *A. duranensis* (90 genes; *AdAPX1-AdAPX90*) and *A. ipaensis* (102 genes; *AiAPX1-AiAPX102*). Their genome sequences were downloaded from PeanutBase database^5^ (Dash et al., 2016). The detailed information (including gene name, gene ID, and protein sequences) of all identified *APX* genes is given in Supplementary Table 1.

Physicochemical features of *AhAPX* were assessed utilizing the ProtParam tool^6^ in the ExPASy server (Gasteiger et al., 2005). Subcellular localization of AhAPX proteins was estimated from CELLO v.2.5^7^ (Yu et al., 2006). Exon-intron configuration of all *AhAPX* were determined using TBtools software (v1.09867)^8^ (Chen et al., 2020). The conserved motifs of AhAPX sequences were documented using the MEME website^9^ (Bailey et al., 2009).

### Evaluation of Chromosomal Location, Phylogenetic Relationships, and Synteny Analysis of *APX* Genes

The data about the chromosomal location of *AhAPX* was attained from the PGR database, and the TBTools was utilized to map the genes on chromosomes. To discover the evolutionary link of the APX proteins, a phylogenetic tree among *A. hypogea* (AhAPXs), *A. duranensis* (AdAPXs), *A. ipaensis* (AiAPXs), and *A. thaliana* (AtAPXs) was created. Multiple sequence alignment was implemented using MEGA7 software^10^ (Kumar et al., 2018). The neighbor-joining (NJ) method was undertaken to design a phylogenetic tree with 1,000 bootstrap replicates and iTOL was used to beautify the tree^11^ (Letunic and Bork, 2021).

The syntenic associations of *APX* genes between *A. hypogea*, *A. duranensis*, *A. ipaensis*, and *A. thaliana* were executed through the MCScanX toolkit and were pictured by the Advance Circos package in the TBTools software (Chen et al., 2020). Additionally, the multiple collinearity analysis of *APX* genes was completed *via* multiple synteny Plot packages in TBTools software. The Ka/Ks ratios of all *AhAPX* were predicted *via* simple Ka/Ks calculator in TBTools software.

### Prediction of *cis*-Regulatory Elements in the *AhAPX* Promoters

To predict the putative *cis*-regulatory elements in the *AhAPX* promoters, the 2 Kb sequences upstream of start codons were separated from the peanut genome. The promoter sequences of all *AhAPX* genes were observed with PlantCARE website^12^ (Lescot et al., 2002), and the picture was illustrated using TBtools software.

### Prediction of Putative miRNAs Targeting *AhAPX* Genes and Functional Annotation Evaluation

The CDS of all *AhAPX* was used to predict the miRNA target sites with psRNATarget website^13^ (Dai et al., 2018) with default considerations. The interactive network figure among the putative miRNAs and *AhAPX* genes was made *via* Cytoscape software (v3.9)^14^ (Shannon et al., 2003). Gene ontology (GO) and Kyoto encyclopedia of genes and genomics (KEGG) annotation evaluation was undertaken by submitting all AhAPX protein sequences to the eggNOG v4.0^15^ (Powell et al., 2014). At the same time, GO and KEGG enrichment evaluations were performed with TBtools software.

### Prediction of Transcription Factor Regulatory Network of *AhAPX* Genes

To predict the putative transcription factors (TFs) and regulatory network, the 500 bp nucleotide sequences from upstream regions of *AhAPX* genes were removed and complied to the PlantRegMap (Transcriptional Regulatory Map)^16^ with *p*-value ≤ 1e^–6^ (Tian et al., 2020). The regulatory network of predicted TFs and *AhAPX* genes was created with Cytoscape v3.9 software.

### Expression Profiling of *AhAPX* Genes

The expression levels of all *AhAPX* genes at diverse developmental tissues/organs (embryo, cotyledon, testa, pericarp, peg, root and stem, root nodule, root tip, root, step tip, stem, leaf, and flower), under various hormones (ethylene, abscisic acid, paclobutrazol, brassinolide, and salicylic acid), and abiotic stress (drought and cold) conditions were evaluated using openly available transcriptome dataset of cultivated peanut (cultivar Shitouqi) at PGR database (see text footnote 1; BioProject PRJNA480120) (Zhuang et al., 2019). The detailed procedure for sample harvesting and data analysis is presented in our recent paper (Zhuang et al., 2019). Owing to the great differences in the expression trends, we normalize the log2 of fragments per kilobase of transcript per million (FPKM) values. Finally, the circular heat maps were designed by TBtools software.

### Plant Material and Stress Conditions

In this study, a widely cultivated peanut variety in southeast China, “Minhua-6” was used for stress treatments. The same variety was also used for transcriptome analysis in our recent paper (Zhuang et al., 2019). The seeds of the “Minhua-6” cultivar were obtained from the FAFU, Fuzhou, China. The vigor seeds were cultured on small pots having a mix of vermicompost. For stress treatment, germinated seedlings at the four-leaf stage were exposed to cold stress at 4°C and ABA (10 μg mL^–1^) for 0 (CK), 3, 6, 9, and 12 h with three biological repetitions. All of the samples were instantly frozen in liquid nitrogen and were kept at -80°C until RNA extraction.

### RNA Extraction and qRT-PCR-based Expression Analysis

Total RNA was isolated utilizing the CTAB method as described in our recent work (Sharif et al., 2021), and cDNA was prepared with the help of Evo M-MLV RT Kit with gDNA Clean for qPCR II (Code No. AG11711; Hunan Aikerui Biological Engineering Co., Ltd., China) following the developer guidelines. The comprehensive information on qRT-PCR reaction has been described in our recent work (Sharif et al., 2021). The peanut *Actin* gene was used as a housekeeping gene to stabilize the expression (Chi et al., 2012). The expression data of three biological repeats were normalized using the 2^–ΔΔCT^ method (Livak and Schmittgen, 2001). All the primers used for qRT-PCR are given in Supplementary Table 2. The graphs were made with GraphPad Prism v9.0.0 software^17^ (Swift, 1997).

## Results

### Comprehensive Characterization of *AhAPX* Genes in Peanut Genome

**FIGURE 1:**
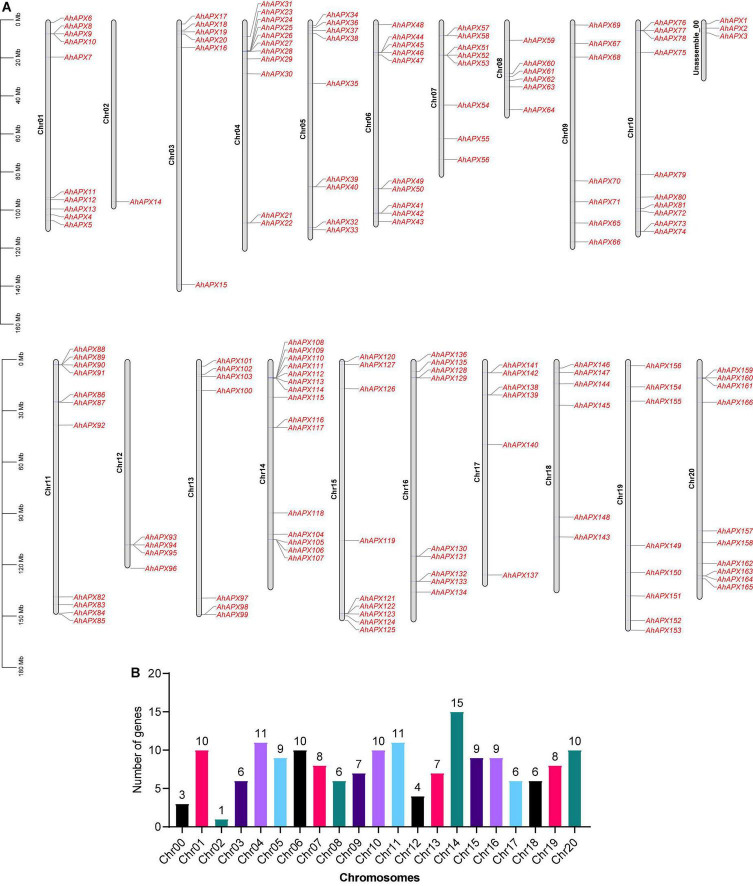
Chromosomal mapping of *AhAPX* genes on *A. hypogea* genome. **(A)** Distribution map of *AhAPX* genes on 20 chromosomes, including one unassembled region. Representative chromosome numbers are shown on the left side of each bar. The scale on the left side represents the chromosomal distance and is in megabases (Mb). **(B)** Graph indicates the number of *AhAPX* genes mapped on each chromosome.

In this study, a total of 166 *AhAPX* genes were discovered in the peanut genome (Table 1). Henceforward, these genes are labeled as “*AhAPX1–AhAPX166*.” These genes were irregularly mapped in the cultivated peanut genome. The maximum number (15) of *AhAPX* genes were mapped on Chr14, followed by Chr04/Chr11 with 11 genes on each chromosome. While, Chr01/Chr06/Chr10/Chr20 were found to have ten genes, followed by Chr05/Chr15/Chr16 with nine genes, Chr07/Chr19 with eight genes, Chr09/Chr13 with seven genes, Chr03/Chr08/Chr17/Chr18 with six genes on each chromosome. The lowest number (1 and 4) of *AhAPX* genes were mapped on Chr02 and Chr12, respectively. Notably, three *AhAPX* genes (*AhAPX1/2/3*) were also mapped on an unassembled region (Chr00) (Figure 1).

**TABLE 1:** The data of 166 *AhAPX* genes identified in peanut genome.

Gene name	Gene ID	Genomic region	CDS length (bp)	Exon	Intron	Protein length (aa)	MW (KDa)	PI	GRAVY	Subcellular localization
*AhAPX1*	AH00G01650.1	Chr00 (2097889, 2103344, +)	1260	11	10	419	44.68	8.64	−0.156	Chloroplast
*AhAPX2*	AH00G03280.1	Chr00 (4356387, 4358168, +)	756	7	6	251	27.79	5.39	−0.491	Cytoplasmic
*AhAPX3*	AH00G04650.1	Chr00 (6939173, 6940468, −)	987	4	3	328	35.75	6.17	−0.223	Extracellular and nuclear
*AhAPX4*	AH01G28620.1	Chr01 (102500998, 102502258, −)	1011	4	3	336	37.9	8.48	−0.37	Nuclear
*AhAPX5*	AH01G31200.1	Chr01 (105607976, 105610045, +)	972	4	3	323	35.1	6.59	−0.101	Extracellular
*AhAPX6*	AH01G01000.1	Chr01 (1376119, 1378806, −)	1005	4	3	334	36.6	8.23	−0.169	Extracellular
*AhAPX7*	AH01G11780.1	Chr01 (19611835, 19615124, +)	981	4	3	326	35.23	8.87	−0.056	Extracellular
*AhAPX8*	AH01G05760.1	Chr01 (7328535, 7331547, +)	969	4	3	322	34.26	6.28	0.029	Extracellular and plasma membrane
*AhAPX9*	AH01G05770.1	Chr01 (7337595, 7340770, −)	987	3	2	328	36.11	9.57	−0.173	Extracellular
*AhAPX10*	AH01G05780.1	Chr01 (7349081, 7354713, −)	969	3	2	322	35.44	8.68	−0.441	Nuclear
*AhAPX11*	AH01G21450.1	Chr01 (93462135, 93464576, +)	987	4	3	328	35.34	6.08	0.017	Extracellular
*AhAPX12*	AH01G22400.1	Chr01 (94528243, 94529833, +)	990	3	2	329	36.09	9.2	−0.206	Extracellular
*AhAPX13*	AH01G26210.1	Chr01 (99439266, 99440500, −)	999	4	3	332	35.7	4.5	−0.139	Extracellular
*AhAPX14*	AH02G25000.1	Chr02 (95622557, 95623515, +)	660	3	2	219	24.43	8.99	−0.35	Extracellular
*AhAPX15*	AH03G45630.1	Chr03 (139181976, 139183516, +)	966	3	2	321	34	6.51	−0.088	Extracellular
*AhAPX16*	AH03G12620.1	Chr03 (14627327, 14629723, +)	978	4	3	325	35.45	6.55	−0.021	Plasma membrane
*AhAPX17*	AH03G01960.1	Chr03 (2186290, 2188245, −)	756	9	8	251	27	5.52	−0.319	Cytoplasmic
*AhAPX18*	AH03G05320.1	Chr03 (5424432, 5426098, +)	978	4	3	325	34.52	8.71	0.015	Extracellular
*AhAPX19*	AH03G06180.1	Chr03 (6345566, 6348916, −)	867	9	8	288	31.66	6.67	−0.311	Cytoplasmic
*AhAPX20*	AH03G07350.1	Chr03 (7481431, 7483098, −)	978	4	3	325	34.61	8.71	0.019	Extracellular and plasma membrane
*AhAPX21*	AH04G21680.1	Chr04 (106761278, 106763236, +)	954	4	3	317	33.53	8.05	−0.061	Extracellular
*AhAPX22*	AH04G21700.1	Chr04 (106776477, 106779460, +)	957	4	3	318	34	4.94	−0.057	Extracellular
*AhAPX23*	AH04G09710.1	Chr04 (16167517, 16170131, +)	912	4	3	303	33.45	7.53	0.003	Extracellular
*AhAPX24*	AH04G09790.1	Chr04 (16461353, 16464502, −)	1068	4	3	355	38.16	6.58	0.005	Extracellular and plasma membrane
*AhAPX25*	AH04G09830.1	Chr04 (16586332, 16594630, −)	1923	7	6	640	69.4	5.78	−0.138	Extracellular and plasma membrane
*AhAPX26*	AH04G09840.1	Chr04 (16603008, 16606152, −)	1113	4	3	370	39.73	5.72	−0.016	Extracellular
*AhAPX27*	AH04G09850.1	Chr04 (16611949, 16615189, −)	1077	4	3	358	38.56	5.74	−0.085	Extracellular
*AhAPX28*	AH04G09870.1	Chr04 (16648330, 16651554, −)	1080	4	3	359	38.83	7.51	−0.154	Extracellular
*AhAPX29*	AH04G10990.1	Chr04 (20506854, 20510294, +)	1047	3	2	348	38.82	5.58	−0.139	Extracellular and plasma membrane
*AhAPX30*	AH04G12400.1	Chr04 (28356898, 28358984, −)	990	4	3	329	36	7.58	−0.1	Extracellular
*AhAPX31*	AH04G06960.1	Chr04 (8749652, 8755948, +)	999	4	3	332	36	5.85	−0.117	Extracellular
*AhAPX32*	AH05G33570.1	Chr05 (109243748, 109247060, +)	1032	4	3	343	37.89	5.3	0.039	Plasma membrane
*AhAPX33*	AH05G34100.1	Chr05 (110343093, 110344607, −)	753	2	1	250	27.34	7.04	−0.191	Cytoplasmic
*AhAPX34*	AH05G02820.1	Chr05 (2952742, 2957237, +)	849	9	8	282	31.57	7.72	−0.496	Cytoplasmic
*AhAPX35*	AH05G12980.1	Chr05 (33401595, 33403392, −)	1269	2	1	422	45.9	5.21	−0.302	Cytoplasmic, nuclear, and extracellular
*AhAPX36*	AH05G03640.1	Chr05 (3964114, 3966683, +)	1047	4	3	348	39.09	5.89	−0.224	Extracellular
*AhAPX37*	AH05G04730.1	Chr05 (5512010, 5518889, −)	984	4	3	327	35.78	9.05	−0.147	Extracellular
*AhAPX38*	AH05G05760.1	Chr05 (7138559, 7139891, −)	984	4	3	327	35.88	6.42	0	Extracellular
*AhAPX39*	AH05G21680.1	Chr05 (87703778, 87704768, +)	645	3	2	214	23.47	5.95	−0.169	Extracellular
*AhAPX40*	AH05G21770.1	Chr05 (87831257, 87833413, −)	984	4	3	327	36	5.5	−0.127	Extracellular
*AhAPX41*	AH06G24710.1	Chr06 (101560772, 101566207, +)	990	3	2	329	35.4	5.31	−0.142	Extracellular and chloroplast
*AhAPX42*	AH06G24750.1	Chr06 (101678350, 101681066, −)	978	3	2	325	35.12	5.87	−0.095	Extracellular
*AhAPX43*	AH06G26990.1	Chr06 (106045942, 106046919, −)	981	1	0	326	35.92	8.33	−0.187	Extracellular and chloroplast
*AhAPX44*	AH06G12580.1	Chr06 (17096141, 17097919, −)	1050	4	3	349	38.3	9.06	−0.146	Extracellular
*AhAPX45*	AH06G12600.1	Chr06 (17107249, 17113857, −)	1923	16	15	640	70.66	8.94	−0.077	Chloroplast
*AhAPX46*	AH06G12640.1	Chr06 (17206186, 17208105, +)	1047	4	3	348	38	9.26	−0.121	Extracellular
*AhAPX47*	AH06G13400.1	Chr06 (18468981, 18470093, −)	957	3	2	318	34.52	8.79	−0.068	Extracellular
*AhAPX48*	AH06G00870.1	Chr06 (2535291, 2536767, −)	981	4	3	326	34.46	5.77	−0.012	Extracellular
*AhAPX49*	AH06G20810.1	Chr06 (88751346, 88752854, −)	954	2	1	317	34.17	9.07	−0.038	Extracellular
*AhAPX50*	AH06G20840.1	Chr06 (88866272, 88866907, −)	639	1	0	212	22.93	6.82	0.017	Chloroplast
*AhAPX51*	AH07G12530.1	Chr07 (18527530, 18532254, −)	972	4	3	323	35	9.76	−0.198	Extracellular and mitochondrial
*AhAPX52*	AH07G12560.1	Chr07 (18594967, 18601313, +)	1005	3	2	334	36.23	5.8	−0.22	Extracellular
*AhAPX53*	AH07G12590.1	Chr07 (18635964, 18639438, −)	900	4	3	299	32.1	6.41	0.088	Plasma membrane
*AhAPX54*	AH07G16820.1	Chr07 (44801964, 44806304, +)	1035	3	2	344	38.1	8.78	−0.11	Plasma membrane
*AhAPX55*	AH07G19820.1	Chr07 (62509939, 62515927, −)	1065	11	10	354	38.47	8.59	−0.324	Mitochondrial
*AhAPX56*	AH07G22100.1	Chr07 (73486525, 73488901, −)	1221	4	3	406	45.08	5.58	−0.359	Nuclear, extracellular, and plasma membrane
*AhAPX57*	AH07G07450.1	Chr07 (8286104, 8288644, −)	975	4	3	324	34.37	8.54	−0.083	Extracellular
*AhAPX58*	AH07G07460.1	Chr07 (8308573, 8309778, +)	762	4	3	253	27.57	8.83	−0.272	Extracellular and mitochondrial
*AhAPX59*	AH08G05850.1	Chr08 (10750010, 10755089, +)	1338	12	11	445	48.75	8.67	−0.428	Chloroplast
*AhAPX60*	AH08G13980.1	Chr08 (28333679, 28334962, −)	756	7	6	251	27.75	5.39	−0.48	Cytoplasmic
*AhAPX61*	AH08G15100.1	Chr08 (29841553, 29842344, −)	795	1	0	264	28.8	4.85	−0.335	Nuclear and cytoplasmic
*AhAPX62*	AH08G16780.1	Chr08 (31925820, 31927225, +)	984	4	3	327	35.85	8.93	−0.108	Extracellular
*AhAPX63*	AH08G19120.1	Chr08 (35244506, 35247372, +)	975	3	2	324	34.42	8.75	−0.044	Extracellular
*AhAPX64*	AH08G26960.1	Chr08 (47233841, 47238063, −)	993	10	9	330	35.63	8.42	−0.221	Chloroplast
*AhAPX65*	AH09G23660.1	Chr09 (106807615, 106809134, +)	984	2	1	327	35.95	8.06	−0.046	Extracellular and mitochondrial
*AhAPX66*	AH09G31660.1	Chr09 (116756835, 116759702, −)	984	4	3	327	35.92	6.09	−0.202	Extracellular
*AhAPX67*	AH09G08990.1	Chr09 (12467446, 12469586, +)	1011	4	3	336	36	9.22	−0.113	Nuclear and plasma membrane
*AhAPX68*	AH09G11440.1	Chr09 (19596211, 19598370, −)	1008	2	1	335	36.05	8.1	−0.094	Chloroplast and extracellular
*AhAPX69*	AH09G02450.1	Chr09 (2707377, 2709690, +)	1365	4	3	454	50.07	4.69	−0.35	Nuclear and plasma membrane
*AhAPX70*	AH09G19280.1	Chr09 (84644615, 84652197, +)	858	4	3	285	31.31	8.41	−0.263	Chloroplast
*AhAPX71*	AH09G20940.1	Chr09 (95646878, 95647778, −)	327	3	2	108	11.59	9.34	−0.157	Extracellular
*AhAPX72*	AH10G22530.1	Chr10 (100608918, 100612037, −)	1038	3	2	345	38.57	5.47	−0.136	Cytoplasmic
*AhAPX73*	AH10G28820.1	Chr10 (111234752, 111235885, −)	969	2	1	322	35.56	6.6	−0.064	Plasma membrane
*AhAPX74*	AH10G28830.1	Chr10 (111237536, 111239429, −)	975	3	2	324	35	5.98	0.028	Plasma membrane
*AhAPX75*	AH10G10440.1	Chr10 (17084135, 17085775, +)	966	4	3	321	35.11	9.49	−0.117	Mitochondrial
*AhAPX76*	AH10G05800.1	Chr10 (5516527, 5517924, −)	981	3	2	326	34.81	7.54	−0.036	Extracellular
*AhAPX77*	AH10G05810.1	Chr10 (5525856, 5527195, +)	996	2	1	331	35.55	8.98	−0.037	Extracellular
*AhAPX78*	AH10G06100.1	Chr10 (5774246, 5774909, −)	594	2	1	197	21	5.61	0.36	Plasma membrane and extracellular
*AhAPX79*	AH10G17560.1	Chr10 (81389354, 81391402, +)	957	3	2	318	3.85	9.4	−0.17	Extracellular
*AhAPX80*	AH10G20050.1	Chr10 (93192984, 93194610, +)	972	4	3	323	34.97	5.46	0.043	Plasma membrane
*AhAPX81*	AH10G21960.1	Chr10 (99235900, 99237538, +)	654	3	2	217	23.87	5.29	0.004	Plasma membrane
*AhAPX82*	AH11G28810.1	Chr11 (138798034, 138799289, +)	1011	4	3	336	37.9	8.48	−0.37	Nuclear
*AhAPX83*	AH11G31650.1	Chr11 (143323830, 143325073, +)	999	4	3	332	35.72	4.41	−0.172	Extracellular
*AhAPX84*	AH11G35510.1	Chr11 (148378457, 148381103, −)	885	3	2	294	32.16	9.23	−0.199	Extracellular
*AhAPX85*	AH11G36340.1	Chr11 (149098252, 149100518, −)	987	4	3	328	35.31	6.07	0.007	Extracellular
*AhAPX86*	AH11G11550.1	Chr11 (24493194, 24496183, +)	981	4	3	326	35.26	8.88	−0.057	Extracellular
*AhAPX87*	AH11G11700.1	Chr11 (25189097, 25190336, +)	687	3	2	228	24.68	4.81	−0.188	Extracellular
*AhAPX88*	AH11G02890.1	Chr11 (3085958, 3089759, −)	984	4	3	327	35.36	8.91	−0.135	Extracellular
*AhAPX89*	AH11G02910.1	Chr11 (3130507, 3135477, +)	1020	3	2	339	37.34	9.07	−0.284	Extracellular
*AhAPX90*	AH11G02940.1	Chr11 (3154813, 3157814, +)	858	3	2	285	31.1	9.36	−0.12	Extracellular
*AhAPX91*	AH11G02950.1	Chr11 (3166698, 3169834, −)	888	5	4	295	31.26	5.06	−0.145	Extracellular
*AhAPX92*	AH11G14080.1	Chr11 (38404886, 38409314, −)	1404	12	11	467	52.19	9.02	−0.453	Nuclear
*AhAPX93*	AH12G26730.1	Chr12 (108404606, 108406854, +)	975	4	3	324	35.2	8.28	−0.002	Extracellular
*AhAPX94*	AH12G26740.1	Chr12 (108408697, 108410986, +)	990	3	2	329	36.09	8.8	−0.234	Extracellular, mitochondrial and chloroplast
*AhAPX95*	AH12G26750.1	Chr12 (108428068, 108430589, +)	987	3	2	328	35.84	5.66	−0.264	Extracellular
*AhAPX96*	AH12G38300.1	Chr12 (122043858, 122045120, +)	996	3	2	331	37.38	6.26	−0.412	Extracellular
*AhAPX97*	AH13G48270.1	Chr13 (139486821, 139487952, +)	966	3	2	321	34	6.51	−0.101	Extracellular
*AhAPX98*	AH13G58440.1	Chr13 (149092750, 149095384, +)	975	3	2	324	35	5.88	0.011	Plasma membrane
*AhAPX99*	AH13G58450.1	Chr13 (149097506, 149098640, +)	969	2	1	322	35.33	6.31	−0.038	Extracellular
*AhAPX100*	AH13G15620.1	Chr13 (18204851, 18207371, +)	978	4	3	325	35.49	8.05	−0.013	Extracellular and plasma membrane
*AhAPX101*	AH13G03790.1	Chr13 (3974837, 3976803, −)	756	9	8	251	27	5.52	−0.319	Cytoplasmic
*AhAPX102*	AH13G08510.1	Chr13 (8737422, 8740756, +)	867	9	8	288	31.66	6.67	−0.311	Cytoplasmic
*AhAPX103*	AH13G09650.1	Chr13 (9945748, 9947398, −)	978	4	3	325	34.5	8.71	0.015	Extracellular
*AhAPX104*	AH14G24560.1	Chr14 (102202325, 102204136, +)	984	4	3	327	35.79	8.44	−0.154	Extracellular
*AhAPX105*	AH14G25410.1	Chr14 (105306662, 105308874, −)	924	4	3	307	32.74	5	−0.086	Extracellular
*AhAPX106*	AH14G25420.1	Chr14 (105316704, 105318852, −)	954	4	3	317	33.51	8.05	−0.034	Extracellular
*AhAPX107*	AH14G25430.1	Chr14 (105327285, 105329671, −)	957	4	3	318	33.64	8.51	−0.084	Extracellular
*AhAPX108*	AH14G08400.1	Chr14 (10636078, 10640476, +)	999	4	3	332	36.11	5.77	−0.137	Extracellular
*AhAPX109*	AH14G08420.1	Chr14 (10696309, 10699909, +)	1068	4	3	355	38.31	6.44	−0.153	Extracellular
*AhAPX110*	AH14G08430.1	Chr14 (10719836, 10722950, +)	1074	4	3	357	38.44	5.74	−0.075	Extracellular
*AhAPX111*	AH14G08440.1	Chr14 (10740576, 10743027, +)	1077	4	3	358	38.25	5.57	0.027	Extracellular
*AhAPX112*	AH14G08450.1	Chr14 (10751673, 10756427, +)	1068	4	3	355	38	4.99	−0.048	Extracellular
*AhAPX113*	AH14G08480.1	Chr14 (10795498, 10798919, +)	1068	4	3	355	38.14	6.2	−0.005	Extracellular
*AhAPX114*	AH14G08550.1	Chr14 (11049580, 11052270, −)	927	4	3	308	33.87	8.05	−0.041	Extracellular
*AhAPX115*	AH14G13430.1	Chr14 (22102438, 22105949, +)	1047	3	2	348	38.8	5.68	−0.149	Extracellular and plasma membrane
*AhAPX116*	AH14G16980.1	Chr14 (39854809, 39855923, +)	570	3	2	189	20.65	6.7	0.287	Extracellular and plasma membrane
*AhAPX117*	AH14G16990.1	Chr14 (39859734, 39860093, +)	363	1	0	120	13.39	9.03	−0.37	Extracellular
*AhAPX118*	AH14G21760.1	Chr14 (89731359, 89733140, −)	909	5	4	302	32.95	8.35	−0.201	Extracellular
*AhAPX119*	AH15G18730.1	Chr15 (105800589, 105804751, −)	1032	4	3	343	37.88	5.3	0.024	Plasma membrane
*AhAPX120*	AH15G00780.1	Chr15 (1195384, 1202015, −)	984	4	3	327	35.78	9.05	−0.147	Extracellular
*AhAPX121*	AH15G33980.1	Chr15 (148489966, 148492117, +)	984	4	3	327	36.17	5.88	−0.164	Extracellular and nuclear
*AhAPX122*	AH15G33990.1	Chr15 (148500369, 148501868, +)	705	4	3	234	25.5	4.8	−0.226	Cytoplasmic and chloroplast
*AhAPX123*	AH15G34130.1	Chr15 (148706618, 148708110, −)	834	5	4	277	30.66	6.22	−0.226	Extracellular and plasma membrane
*AhAPX124*	AH15G35170.1	Chr15 (149938106, 149940460, −)	669	3	2	222	24.22	7.67	−0.021	Extracellular
*AhAPX125*	AH15G37650.1	Chr15 (152607618, 152611276, −)	1092	3	2	363	40.13	5.51	−0.222	Extracellular
*AhAPX126*	AH15G09760.1	Chr15 (17115019, 17116810, +)	1257	2	1	418	45.61	5.4	−0.337	Extracellular, cytoplasmic and nuclear
*AhAPX127*	AH15G01790.1	Chr15 (3022442, 3024001, −)	984	4	3	327	36	6.87	−0.042	Extracellular
*AhAPX128*	AH16G05890.1	Chr16 (10586280, 10589926, −)	987	4	3	328	35.89	7.97	−0.131	Extracellular
*AhAPX129*	AH16G06030.1	Chr16 (10824424, 10825859, +)	882	4	3	293	32.28	8.53	−0.268	Extracellular
*AhAPX130*	AH16G25780.1	Chr16 (114921710, 114923167, −)	954	2	1	317	33.99	8.98	−0.027	Extracellular
*AhAPX131*	AH16G25800.1	Chr16 (115100883, 115101305, −)	426	1	0	141	15.52	6.28	−0.05	Extracellular, cytoplasmic and chloroplast
*AhAPX132*	AH16G30440.1	Chr16 (129635392, 129640099, +)	990	3	2	329	35.36	5.32	−0.127	Extracellular and chloroplast
*AhAPX133*	AH16G30490.1	Chr16 (129750891, 129753522, −)	975	3	2	324	34.81	5.87	−0.047	Extracellular
*AhAPX134*	AH16G33620.1	Chr16 (135979748, 135980725, −)	981	1	0	326	35.95	8.69	−0.161	Extracellular
*AhAPX135*	AH16G03520.1	Chr16 (7153751, 7155209, −)	981	4	3	326	34.38	5.31	−0.009	Extracellular
*AhAPX136*	AH16G01030.1	Chr16 (993418, 995239, +)	1050	4	3	349	38.29	9.17	−0.139	Extracellular
*AhAPX137*	AH17G30310.1	Chr17 (125992323, 125997218, −)	1338	12	11	445	48.73	8.8	−0.44	Chloroplast
*AhAPX138*	AH17G11990.1	Chr17 (20654955, 20659787, −)	972	4	3	323	34.93	9.74	−0.18	Extracellular
*AhAPX139*	AH17G12030.1	Chr17 (20764144, 20766484, +)	735	2	1	244	26.45	6.81	−0.317	Extracellular
*AhAPX140*	AH17G18150.1	Chr17 (49822929, 49829110, +)	1068	11	10	355	38.64	8.79	−0.338	Mitochondrial
*AhAPX141*	AH17G06310.1	Chr17 (7780769, 7782202, −)	963	4	3	320	34.92	8.65	−0.145	Extracellular
*AhAPX142*	AH17G06350.1	Chr17 (7839321, 7842337, +)	966	4	3	321	34	8.54	−0.113	Extracellular
*AhAPX143*	AH18G23730.1	Chr18 (103745127, 103747854, +)	1014	3	2	337	37.31	9.13	−0.136	Mitochondrial and plasma membrane
*AhAPX144*	AH18G10570.1	Chr18 (14253327, 14256241, +)	972	3	2	323	34.3	8.75	−0.044	Extracellular
*AhAPX145*	AH18G15530.1	Chr18 (26788797, 26795823, −)	1416	16	15	471	52.82	9	−0.639	Nuclear
*AhAPX146*	AH18G05400.1	Chr18 (5046262, 5048240, −)	969	2	1	322	35.34	5.8	−0.104	Plasma membrane
*AhAPX147*	AH18G07180.1	Chr18 (7610724, 7612104, +)	987	4	3	328	35.93	8.82	−0.101	Extracellular
*AhAPX148*	AH18G22460.1	Chr18 (92176526, 92178893, +)	1218	4	3	405	45.05	5.81	−0.101	Extracellular, nuclear and plasma membrane
*AhAPX149*	AH19G24230.1	Chr19 (108739539, 108750043, −)	996	4	3	331	36.82	6.95	−0.132	Extracellular
*AhAPX150*	AH19G26520.1	Chr19 (124514590, 124517216, −)	996	4	3	331	40.26	7.97	0	Extracellular and chloroplast
*AhAPX151*	AH19G29790.1	Chr19 (138198709, 138200248, +)	984	2	1	327	35.98	8.06	−0.054	Extracellular and mitochondrial
*AhAPX152*	AH19G36370.1	Chr19 (152526370, 152529191, +)	984	4	3	327	36.19	5.9	−0.225	Extracellular and nuclear
*AhAPX153*	AH19G42570.1	Chr19 (158270903, 158272107, −)	975	3	2	324	35.69	9.04	−0.179	Extracellular
*AhAPX154*	AH19G11940.1	Chr19 (16033083, 16035651, +)	1014	4	3	337	36.06	8.91	−0.113	Extracellular and plasma membrane
*AhAPX155*	AH19G14960.1	Chr19 (24411814, 24415355, −)	1278	3	2	425	46.49	6.47	−0.117	Chloroplast
*AhAPX156*	AH19G03800.1	Chr19 (3648102, 3650393, +)	1365	4	3	454	49.95	4.72	−0.351	Nuclear
*AhAPX157*	AH20G22440.1	Chr20 (100242945, 100244252, −)	987	4	3	328	35.74	6.17	−0.213	Extracellular and nuclear
*AhAPX158*	AH20G23580.1	Chr20 (107122955, 107124841, +)	957	3	2	318	33.85	9.4	−0.173	Extracellular
*AhAPX159*	AH20G08720.1	Chr20 (10757292, 10758717, −)	981	3	2	326	34.71	6.07	−0.028	Extracellular
*AhAPX160*	AH20G08730.1	Chr20 (10773674, 10775017, +)	999	2	1	332	35.59	8.98	−0.023	Extracellular
*AhAPX161*	AH20G09010.1	Chr20 (11124003, 11124708, −)	636	2	1	211	22.75	4.93	0.392	Plasma membrane
*AhAPX162*	AH20G26300.1	Chr20 (119159975, 119161601, +)	972	2	1	323	35	5.32	0.067	Plasma membrane
*AhAPX163*	AH20G28750.1	Chr20 (126510516, 126511240, +)	465	2	1	154	17	6.4	−0.372	Nuclear
*AhAPX164*	AH20G28770.1	Chr20 (126544791, 126546815, +)	927	4	3	308	33.85	4.89	−0.132	Extracellular and cytoplasmic
*AhAPX165*	AH20G29320.1	Chr20 (128237247, 128240109, −)	1038	3	2	345	38.54	5.38	−0.138	Cytoplasmic
*AhAPX166*	AH20G14810.1	Chr20 (25055084, 25056732, +)	969	4	3	322	35.15	9.54	−0.139	Mitochondrial

*In the genomic position, the positive (+) and negative (−) sign shows the presence of a gene on the positive and negative strand of that specific marker correspondingly. MW, molecular weight; PI, isoelectric points; bp, base pair; aa, amino acids.*

Comprehensive information of all predicted 166 *AhAPX* genes is presented in Table 1. In short, the CDS length varied from 327 bp (*AhAPX71*) to 1,923 bp (*AhAPX25/45*), and the amino acid length assorted from 108 (*AhAPX71*) to 640 (*AhAPX25/45*) amino acids. The number of exons varied from one (*AhAPX43/50/61/117/131/134*) to 16 (*AhAPX45/145*) (Table 1). Particularly, only two genes (*AhAPX45* and *AhAPX145*) had the uppermost number of introns (i.e., 15), and quite a few genes lack introns (i.e., *AhAPX43/50/61/117/131/134*) (Table 1). The anticipated molecular weights (MW) of the 166 AhAPX proteins increased from 3.85 kDa (AhAPX79) to 70.66 kDa (AhAPX45), the isoelectric points (PI) extended from 4.41 (AhAPX83) to 9.76 (AhAPX51), and the GRAVY ranged from -0.639 (AhAPX145) to 0.392 (AhAPX161). The transformations in MW and PI are primarily due to the elevated content of necessary amino acids and post-translational alterations. The *in silico* subcellular localization discovered that 115 AhAPX proteins were situated on the extracellular matrix, 14 AhAPX proteins on plasma membrane, 12 AhAPX proteins on cytoplasm, nine AhAPX proteins on chloroplast, and five AhAPX proteins on mitochondrion (Table 1). Notably, some AhAPX proteins were found to be located in more than one location (Table 1).

On the other hand, eight genes (*AtAPXs*) from *A. thaliana*, 90 genes (*AdAPX1-AdAPX90*) from *A. duranensis*, and 102 genes (*AiAPX1-AiAPX102*) from *A. ipaensis* genomes were also recognized to study the evolution of *APX* genes between tetraploid and diploid parents (Supplementary Table 1).

### Insights From Phylogenetic Relationships of *APX* Proteins

**FIGURE 2:**
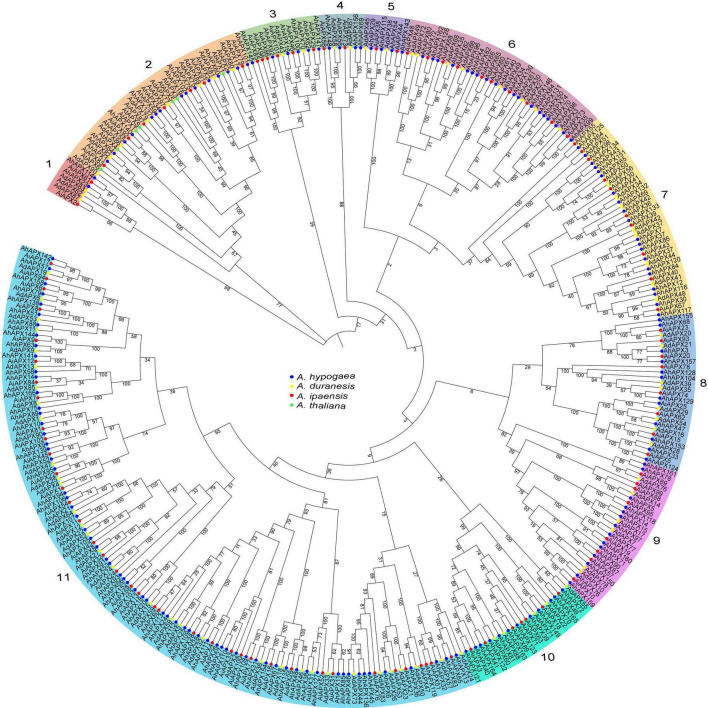
An unrooted neighbor-joining phylogenetic tree of APX proteins from *A. hypogea, A. duranensis, A. ipaensis*, and *A. thaliana*. On the whole, 166 AhAPXs from *A. hypogea* (blue circles), 90 AdAPXs from *A. duranensis* (yellow circles), 102 AiAPXs from *A. ipaensis* (red circles), and 8 AtAPXs from *Arabidopsis thaliana* (green circles) were clustered into 11 groups based on sequence similarities, domain, and 1,000 bootstrap values. The percentage of bootstrap values is shown in the notes.

To determine the in-depth evolutionary and phylogenetic history between the AhAPX (166 members), AdAPX (90 members), AiAPX (102 members), and AtAPX proteins (8 members), an unrooted phylogenetic tree was built by a multiple sequence alignment, which was divided into 11 main groups (group1–group11) (Figure 2). The discoveries exposed that group1 comprised of seven APX members (2 AhAPX, 2 AiAPX, and 3 AdAPX) followed by group4/5 (eight APX members), and group3 (14 APX members). Notably, the maximum number of APX members (66 AhAPX, 39 AiAPX, and 33 AdAPX) were found in group 11 followed by group7 (38 APX members), group2/6 (37 APX members), group8/9 (28 APX members), and group10 (24 APX members) (Figure 2). All AtAPX members were clustered only in one group, i.e., group 2. In general, APXs grouped into the indistinguishable sub-group may retain corresponding functions. It is worth stating that *A. hypogea* APX (AhAPXs) were distributed in each group with homologs from *A. duranensis, A. ipaensis*, and *A. thaliana*., and group11 was detected to have more AhAPX members than the other 10 groups (Figure 2). Furthermore, it was observed that the AhAPXs showed a greater phylogenetic network with the AdAPXs and AiAPXs in each group.

### Insights Into Synteny and Collinearity of *APX* Genes

**FIGURE 3:**
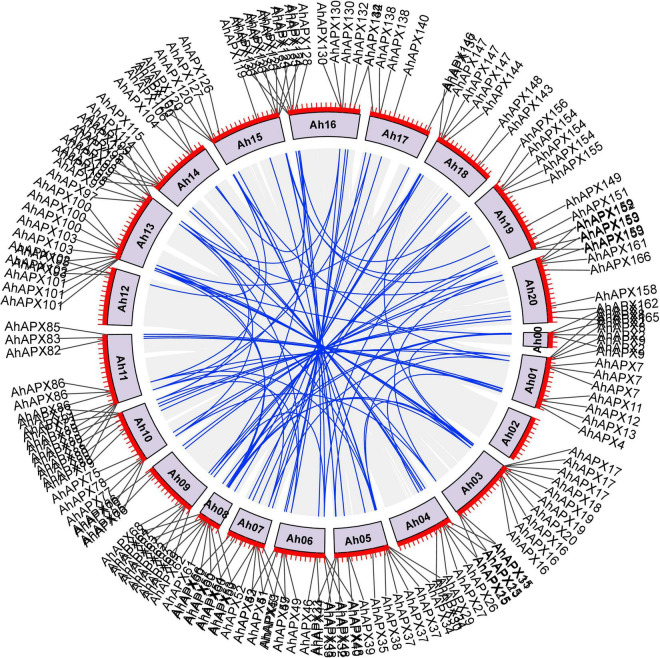
Chromosomal positions and inter-chromosomal groups of duplicated *AhAPX* gene pairs. Gray lines in the background demonstrate all syntenic blocks in the *A. hypogea* genome, and the blue lines exhibit the segmental or tandem duplication network zones among *AhAPX* genes. The near location of *AhAPX* genes is marked with black lines outside with chromosome names.

Gene duplications (i.e., tandem and segmental) are thought to be the main factors in supporting the expansion and evolution of new gene families in plants (Cannon et al., 2004). Hence, gene duplication procedures were assessed between *AhAPXs, AdAPXs, AiAPXs*, and *AtAPXs* (Supplementary Table 3). The results of gene duplication study showed that there were 92 *AhAPX* gene pairs, and these pairs were unevenly mapped on different chromosomes (Figure 3 and Supplementary Table 3). Mainly, chromosome 13 had a maximum number (i.e., 16) of *AhAPX* gene pairs, followed by chromosome 5 with 12 *AhAPX* gene pairs. The least number of gene pairs (i.e., two) was discovered on chromosome 12, and no gene pair was found on chromosome 2 (Figure 3 and Supplementary Table 3). The results reveal that segmental duplications have contributed to the expansion of *AhAPX* genes in the cultivated peanut genome (Supplementary Table 3). Notably, no tandem duplicated gene pairs were identified.

Similarly, 10 duplicated gene pairs were detected between *AhAPX* and *AtAPX* (Supplementary Figure 1 and Supplementary Table 3); 171 pairs between *AhAPX* and *AiAPX* (Supplementary Figure 2 and Supplementary Table 3); and 160 pairs between *AhAPX* and *AdAPX* (Supplementary Figure 3 and Supplementary Table 3). All these gene pairs were irregularly mapped on different chromosomes. Taken together, these conclusions explained that the duplication activities played a vital role in enlarging the *APX* genes between diploid and tetraploid parents. Further, it can also be concluded that *A. hypogea* might have lost some genes during genome evolution.

**FIGURE 4:**
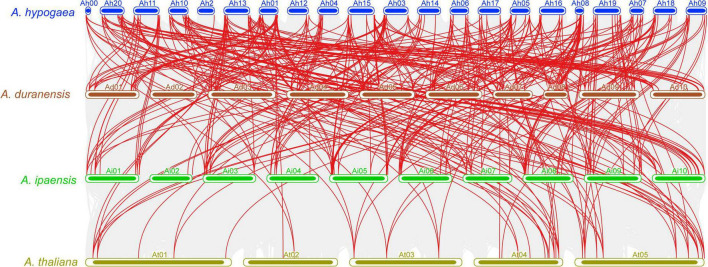
Multiple collinearity analysis of *APX* genes between *A. hypogea, A. duranensis, A. ipaensis*, and *A. thaliana* chromosomes. Gray lines in the background specify the collinear blocks within *A. hypogea* and other three genomes, while the red lines highlight the syntenic *APX* orthologous gene pairs.

Collinearity analysis was carried out to review the evolutionary association of the *APX* genes between *A. hypogea, A. duranensis, A. ipaensis*, and *A. thaliana* (Figure 4 and Supplementary Table 3). The results discovered a strong orthologous of *APX* genes among these four species (Figure 4). On the whole, several *A. hypogea* genes presented syntenic networks with different *AdAPX, AiAPX*, and *AtAPX* genes. Particularly, only one gene (*AhAPX14*) at chromosome Ah2 exhibited a syntenic connection with *AdAPX85* gene at chromosome Ad02 (Figure 4 and Supplementary Table 3), while other homologous genes present on other *A. hypogea* chromosomes also showed a syntenic relationship with many *AdAPX*, *AiAPX* and *AtAPX* genes (Figure 4 and Supplementary Table 3). These findings indicate that whole-genome or segmental duplication procedures are considered a main evolutionary force in the evolution of *AhAPX* genes in the peanut genome (Figure 4 and Supplementary Table 3).

The Ka/Ks ratio is considered as a huge diagnostic marker in evaluating the sequence evolution in terms of selection pressures and duplication types (Hurst, 2002). Thus, to understand the evolutionary story of the *AhAPX*, the Ka, Ks, and Ka/Ks ratio was revealed (Supplementary Table 3). The dataset unveiled that all duplicated *AhAPX* gene pairs had a Ka/Ks ratio of <1 (Supplementary Table 3), demonstrating that the *AhAPX* genes may have experienced strong purifying selective pressure and segmental duplications throughout the evolution procedure (Supplementary Table 3).

### Insights Into Gene Structures and Conserved Motifs of *AhAPX* Genes

**FIGURE 5:**
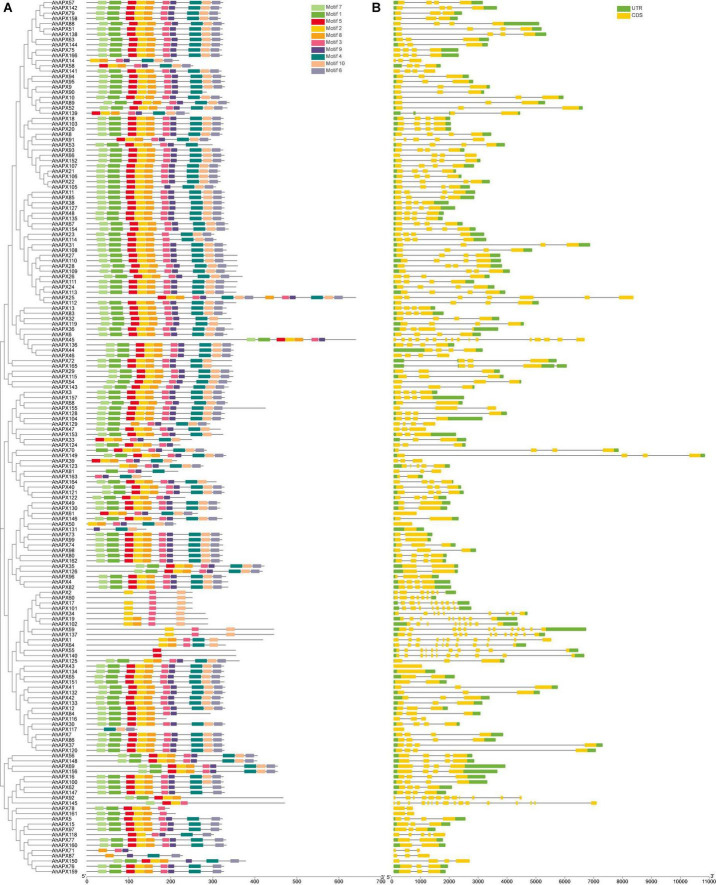
The gene structure and motif analysis of the *AhAPX* genes. **(A)** Arrangements of predicted conserved motifs in *AhAPXs*. Unique color boxes exhibit individual motifs. **(B)** The gene structure of the *AhAPXs*. Light green color indicates the UTR regions; yellow color shows the CDS regions, and gray horizontal line displays the introns.

The exon-intron arrangements and conserved motifs of the *AhAPX* genes were analyzed to get insights into the advancement of the *APX* family genes in peanut genome (Figure 5 and Supplementary Table 4). The outcomes revealed that the number of exons and introns varied from 16 to 1 and 0 to 15, respectively (Figure 5B and Supplementary Table 10). In short, 6 genes have 1 exon and zero intron; 13 genes have 2 exons and 1 intron; 5 genes have 3 exons and 2 introns; 18 genes have 2 exons and 1 intron; 41 genes have 3 exons and 2 introns; 81 genes have 4 exons and 3 introns; 3 genes have 5/7 exons and 4/6 introns; 5 genes have 9 exons and 8 introns; only 1 gene has 10 exons and 9 introns; 3 genes have 11/12 exons and 10/11 introns; and only 2 genes have a maximum number of exons (16) and introns (15) (Figure 5B and Supplementary Table 10). Above all, genes belonging to the same sub-tree almost had parallel structures apart from a few genes (Figure 5B). Among all genes, *AhAPX149* possess the longest structure, and only a few genes have a complex structure, such as *AhAPX17, AhAPX19, AhAPX34, AhAPX45, AhAPX55, AhAPX59, AhAPX64, AhAPX92, AhAPX101, AhAPX102*, and *AhAPX145* (Figure 5B). Exon loss or gain has been found during the evolution of *APX* family genes. The results recommended that *APX* genes held a somewhat frequent exon-intron composition throughout the evolution of peanut genome. Furthermore, *AhAPX* gene participants inside a sub-tree had exceptionally corresponding gene structures, steady with their phylogenetic clusters.

The conserved motif of the *AhAPX* genes ranged from one (*AhAPX55/140*) to three (*AhAPX2/71/117/131*) (Figure 5A). In total, 10 conserved motifs were recognized, and their complete dataset, including motif names, sequences, width, and E-value, is given in Supplementary Table 4. Similar to gene structure, the motif distributions were also similar within the sub-trees (Figure 5A), while some motifs were found to be specific to some genes. For instance, some genes such as *AhAPX2/17/101/34/59/137* were limited to motifs 2, 3, and 10. While *AhAPX60* gene only contained motifs 3 and 10; AhAPX71 contained motifs 3, 8, and 9; AhAPX117 contained motifs 4, 6, and 10; AhAPX131 contained motifs 4, 9, and 10; and AhAPX71 contained motifs 3, 8, and 9 (Figure 5A). Almost all other motifs were present on all genes except in a few cases (Figure 5A). In summary, the consistency of gene organizations within sub-trees was credibly constant by appraising the conserved motif structures, gene structures, and phylogenetic relations, representing that the APX proteins have enormously well-sustained amino acid deposits and APX members belonging to the same tree may hold corresponding roles.

### *Cis*-Elements: Key Players in the Promoter Regions of *AhAPX* Genes

**FIGURE 6:**
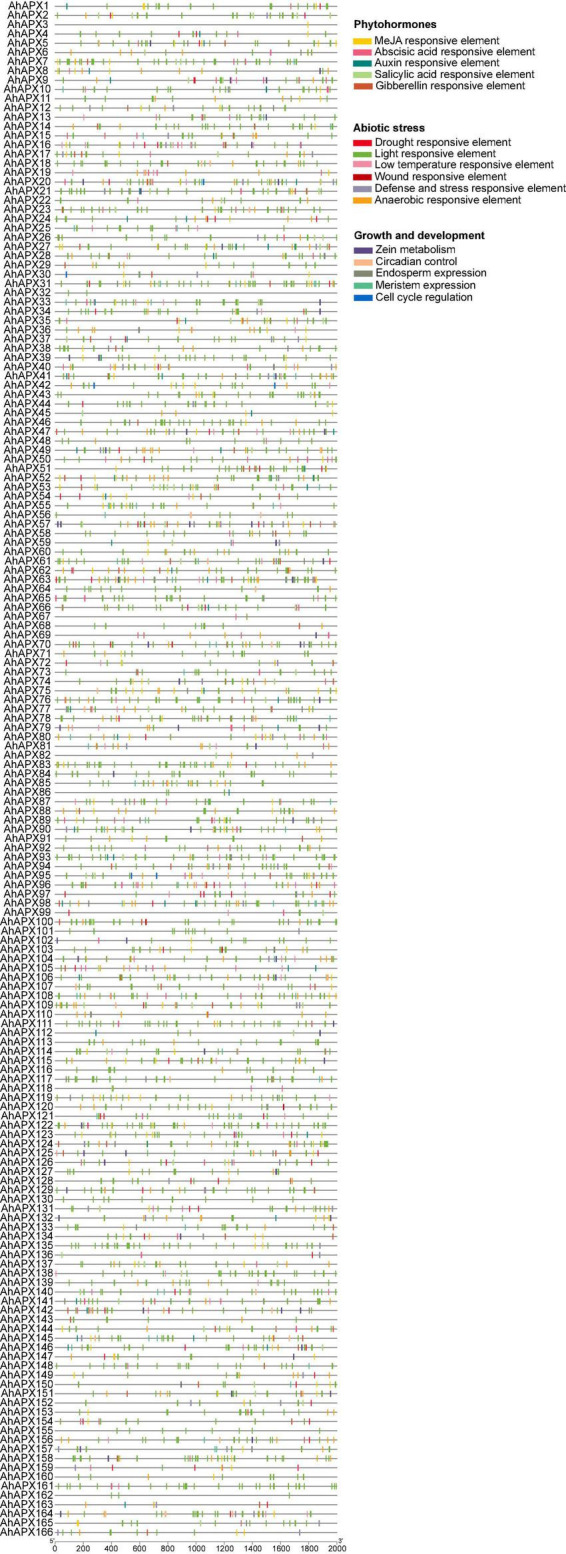
Analysis of *cis*-regulatory elements in the *AhAPX* promoter regions. Diverse *cis*-elements with functional resemblance are represented by similar colors.

**FIGURE 7:**
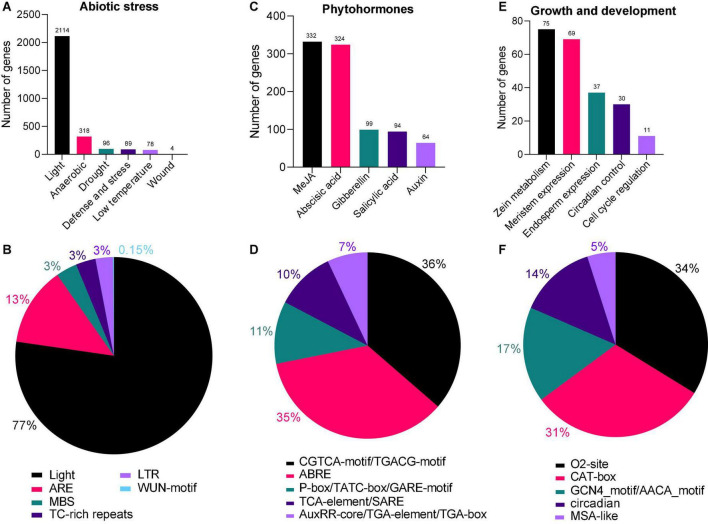
*Cis*-regulatory elements in the *AhAPX* promoter regions. **(A,C,E)** The total number of *AhAPX* genes involved in abiotic stress, phytohormones, and growth and development categories of *cis*-elements. **(B,D,F)** The percentage (%) ratio of the several *cis*-elements from each category is shown in pie charts, i.e. **(B)** abiotic stress-responsive, **(D)** phytohormones responsive, and **(F)** plant growth and development responsive. Diverse colors signify different *cis*-acting elements and their ratio present in *AhAPX* genes.

To better understand the regulatory role of *AhAPX* genes toward peanut growth and development, and tolerance to abiotic stress and phytohormones treatment, *cis*-regulatory elements in the promoter of *AhAPX* were explored. The complete dataset of *cis*-elements is presented in Supplementary Table 5. We emphasized and recognized three categories of *cis*-elements, including abiotic stress-responsive, phytohormones responsive, and growth and development responsive elements (Figures 6, 7 and Supplementary Table 5). Mainly, six abiotic stress-responsive (drought, light, low temperature, wound, defense and stress, and anaerobic) elements were detected. These elements consist of I-box, ATCT-motif, Box 4, GT1-motif, GA-motif, etc. (light-responsive, 77%), ARE (13%), MBS (3%), TC-rich repeats (3%), LTR (3%), and WUN-motif (0.15%) (Figures 7A,B and Supplementary Table 5). Overall, results showed that most of the abiotic stress-related elements were predicted to be specific to some genes and unevenly distributed (Figure 6 and Supplementary Table 5), indicating their defensive role against stress conditions.

Likewise, five phytohormone-responsive elements [methyl jasmonate (MeJA), abscisic acid, gibberellin, salicylic acid, and auxin] consist of CGTCA-motif/TGACG-motif (36%), ABRE (35%), P-box/TATC-box/GARE-motif (11%), TCA-element/SARE (11%), and AuxRR-core/TGA-element/TGA-box (7%) (Figures 7C,D and Supplementary Table 5). Some of the elements were found to be specific to some genes and unevenly distributed (Figure 6 and Supplementary Table 5). These outcomes suggest that element-specific genes could be considered as candidate players for further functional studies to reveal their protective role under hormone treatments.

Moreover, five growth and development-related (zein metabolism, meristem expression, endosperm expression, circadian control, and cell cycle regulation) elements were discovered. These key elements include O_2_-site (34%), CAT-box (31%), GCN4_motif/AACA_motif (17%), circadian (14%), and MSA-like (5%) (Figures 7E,F and Supplementary Table 5), suggesting their dynamic role in different growth and developmental stages of peanut. In a nutshell, these discoveries suggested that some of the key elements are widely and randomly distributed in some genes, while some of the elements are found to be specific to some genes. It can be concluded that the expression profiles of *AhAPX* genes may fluctuate under different developmental stages, phytohormone and abiotic stress conditions.

### Genome-Wide Investigation of miRNAs Targeting *AhAPX* Genes

**FIGURE 8:**
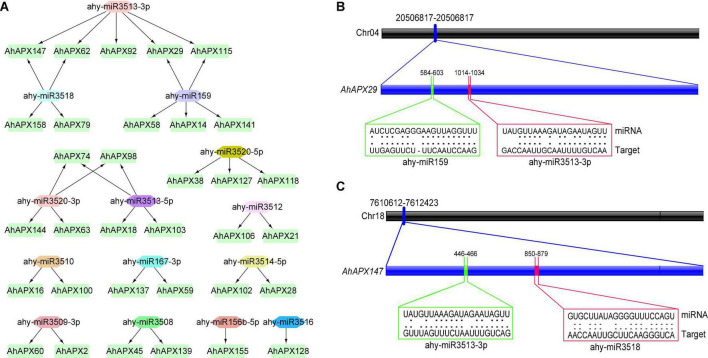
miRNA targeting *AhAPX* genes. **(A)** Network map of projected miRNA targeting *AhAPX* genes. The green boxes correspond to *AhAPX* genes, and various color shapes indicate predicted miRNAs. **(B)** The graphic illustration indicates the *AhAPX29* gene is targeted by miRNAs (ahy-miR159 and ahy-miR3513-3p). **(C)** The graphic illustration shows the *AhAPX147* gene is targeted by miRNAs (ahy-miR3518 and ahy-miR3513-3p). Black thick bar indicates the chromosomal location of gene. Blue thick bar indicates the location of miRNAs on gene sequence. The RNA sequence of each complementary site from 5′ to 3′ and the predicted miRNA sequence from 3′ to 5′ are shown in the green and red line boxes. The complete dataset of predicted miRNAs is shown in Supplementary Table 6.

To better comprehend the miRNA-arbitrated post-transcriptional alteration of *AhAPX* genes, we identified 14 miRNAs targeting 33 genes (Figure 8A and Supplementary Table 6). These miRNAs belong to 12 different families. To give an overview, the miRNA-targeted sites of *AhAPX29* and *AhAPX147* are shown in Figures 8B,C, whereas the complete dataset of all miRNAs targeted sites/genes is provided in Supplementary Table 6. The results showed that ahy-miR159 and ahy-miR3513-3P targeted the most number (5) of genes. Three miRNAs, including ahy-miR3518, ahy-miR3520-3P, and ahy-miR3513-5P targeted four genes, followed by ahy-miR3520-5P that targeted three genes (*AhAPX38*, *AhAPX127*, and *AhAPX118*). While six miRNAs including ahy-miR3512, ahy-miR3510, ahy-miR167-3P, ahy-miR3514-5P, ahy-miR3509-3P, and ahy-miR3508 targeted two different genes individually. Notably, only two miRNAs (ahy-miR156b-5p and ahy-miR3516) targeted one gene, *AhAPX155* and *AhAPX128*, respectively (Figure 8A and Supplementary Table 6). Some common genes like *AhAPX29, AhAPX62, AhAPX115, AhAPX147, AhAPX74*, and *AhAPX98* are found to be targeted by more than one miRNA. Hence, the expression profiling of these predicted miRNAs and their targeted genes necessitates confirmation to oversee their biological roles in the cultivated peanut genome.

### Transcription Factor Regulatory Network of *AhAPX* Genes

**FIGURE 9:**
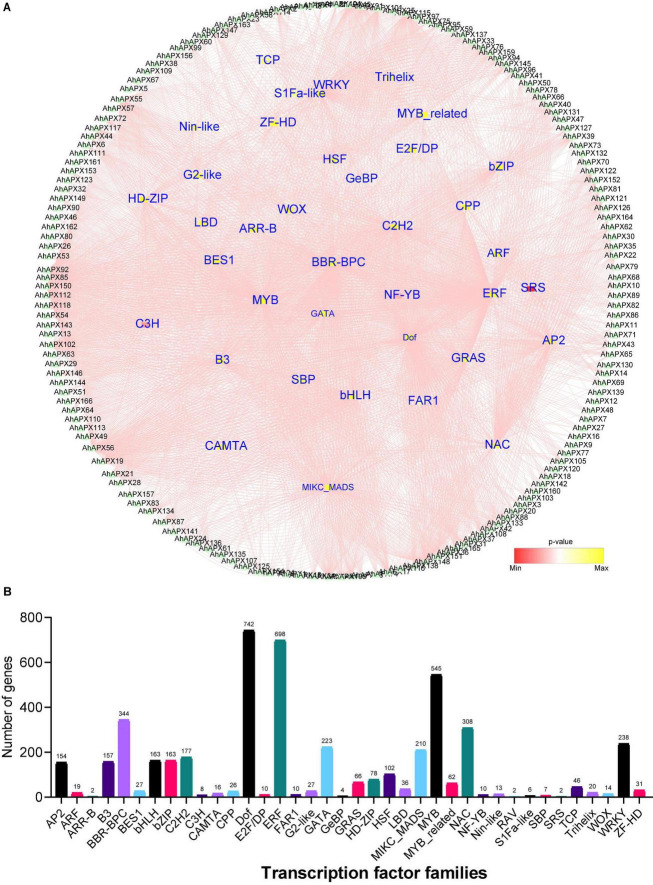
The regulatory network of putative transcription factors of *AhAPX* genes. **(A)** Circular network of transcription factors. *AhAPX* genes are shown around the circle. The small inner circles denote the transcription factors, and their color pink to yellow represents the degree of interaction. **(B)** Graph indicates the number of *AhAPX* genes and their representative putative transcription factor families. The complete dataset of putative transcription factors is shown in Supplementary Table 7.

To get further insights into the regulatory role of transcription factors (TFs) in regulating the transcription of *AhAPX* genes, we identified 3,257 TFs in 162 *AhAPX* genes (Figure 9 and Supplementary Table 7). The results showed that these TFs belong to 38 diverse TFs families, including AP2, ARF, B3, bHLH, bZIP, Dof, ERF, MYB, NAC, WRKY, HSF, GATA, etc. (Figure 9 and Supplementary Table 7). The amplest TFs families were Dof (742 members), ERF (698 members), MYB (545 members), BBR-BPC (344 members), NAC (308 members), WRKY (238 members), GATA (223 members), MIKC_MADS (210 members), C2H2 (177 members), bHLH/bZIP (163 members), B3 (157 members), AP2 (154 members), and HSF (102 members) (Figure 9B and Supplementary Table 7). However, the least ample TFs families were ARR-B/RAV/SRS (2 members), followed by GrBP (4 members), S1Fa-like (6 members), SBP (7 members), C3H (8 members), etc. (Figure 9B and Supplementary Table 7). In contrast, other TFs families contained less than 100 members. Nearly, all 162 *AhAPX* genes were anticipated to be targeted by various TFs belonging to diverse families. For instance, *AhAPX150* gene was abundantly tarted by 314 TFs, followed by *AhAPX56* by 172 TFs, *AhAPX148* by 145 TFs, *AhAPX55* by 107 TFs, *AhAPX92* by 93 TFs, *AhAPX45* by 96 TFs., etc. (Figure 9 and Supplementary Table 7). Some genes were nominally targeted, e.g., *AhAPX5/79/99* by 1 TF, *AhAPX83/90/93* by 2 TFs, *AhAPX9/15* by 3 TFs, *AhAPX7* by 4 TFs, *AhAPX8/70/80* by 5 TFs., etc. (Figure 9 and Supplementary Table 7). Overall, these results showed that abiotic and phytohormone-related TFs could be engineered to develop improved peanut cultivars.

### Gene Ontology and Kyoto Encyclopedia of Genes and Genomics Enrichment Analysis of *AhAPX* Genes

**FIGURE 10:**
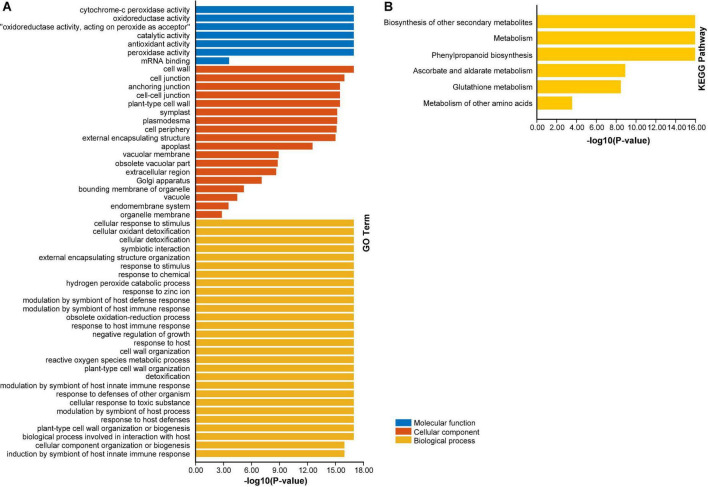
Gene ontology (GO) and KEGG enrichment analysis of *AhAPX* genes. **(A)** The highly enriched GO terms from MF, CC, BP classifications in *AhAPX* genes. **(B)** The highly enriched KEGG pathways in *AhAPX* genes.

To advance our knowledge about the dynamic roles of *AhAPX* genes at molecular level, GO and KEGG enrichment analysis were performed (Figure 10 and Supplementary Table 8). The GO annotation outcomes of biological process (BP), molecular function (MF), and cellular component (CC) classes presented quite a few substantially enriched terms (Figure 10A and Supplementary Table 8). For instance, in MF class, the highly enriched terms were cytochrome-c peroxidase activity (GO:0004130), oxidoreductase activity (GO:0016491), catalytic activity (GO:0003824), antioxidant activity (GO:0016209), and peroxidase activity (GO:0004601). In CC class, the most enriched terms were cell wall (GO:0005618), and cell junction (GO:0030054). Whereas in BP class, the highly enriched terms were cellular response to stimulus (GO:0051716), cellular detoxification (GO:1990748), response to chemical (GO:0042221), hydrogen peroxide catabolic process (GO:0042744), response to zinc ion (GO:0010043), modulation by symbiont of host defense response (GO:0052031), obsolete oxidation-reduction process (GO:0055114), detoxification (GO:0098754)., etc. (Figure 10A and Supplementary Table 8).

Furthermore, KEGG pathway enrichment study discovered six pathways participating in diverse functions of *AhAPX* genes (Figure 10B and Supplementary Table 8). The highly enriched pathways include biosynthesis of other secondary metabolites (B09110), metabolism (A09100), phenylpropanoid biosynthesis (00940), followed by ascorbate and aldarate metabolism (00053), glutathione metabolism (00480), and metabolism of other amino acids (B09106) (Figure 10B and Supplementary Table 8). Briefly, it can be concluded that GO and KEGG enrichment study validates the functional contribution of *AhAPX* genes in several cellular, molecular, and biological processes, that are associated with antioxidant defense systems, ROS scavenging, response to stresses, and biosynthesis of different metabolites.

### Expression Profiling of *AhAPX* Genes at Diverse Developmental Tissues

**FIGURE 11:**
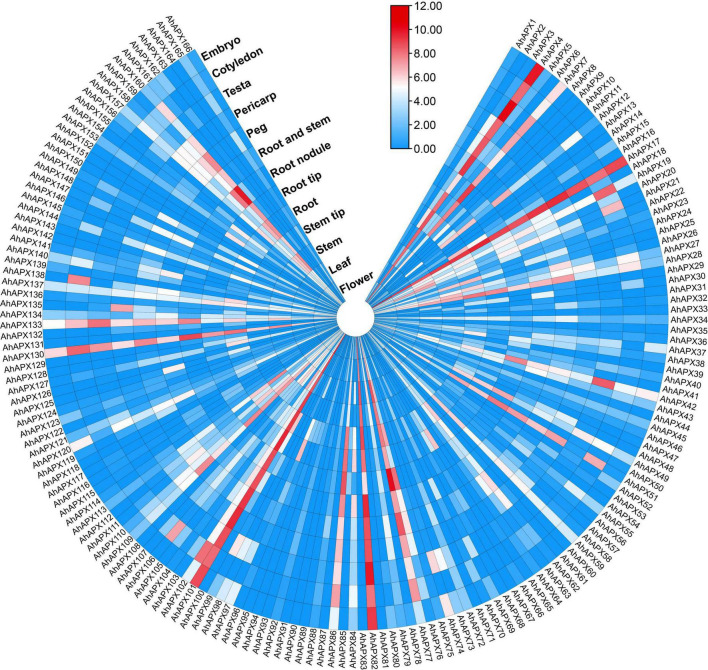
Expression profiling of *AhAPX* genes in various developmental tissues of peanut. In the expression bar, the red, white and blue colors show high to low expression levels. The circular heat map was designed by taking log2 of fragments per kilobase of transcript per million (FPKM) values.

The expression profiling of 166 *AhAPX* genes was observed in various tissues and organs, including embryo, cotyledon, testa, pericarp, peg, root and stem, root nodule, root tip, root, step tip, stem, leaf, and flower using openly available transcriptome dataset (Supplementary Table 9). Overall, the expression heatmap indicated that only a few genes were highly expressed in certain organs/tissues (Figure 11 and Supplementary Table 9). For example, some genes including *AhAPX4, AhAPX7, AhAPX17, AhAPX19, AhAPX28, AhAPX42, AhAPX51, AhAPX76, AhAPX77, AhAPX82, AhAPX86, AhAPX101, AhAPX102, AhAPX130, AhAPX133*, and *AhAPX160* were highly expressed in almost all the organs/tissues (Figure 11). While some genes were found to be specific to some tissues like *AhAPX12* showed considerable expression in cotyledon, root and stem, root tip, and stem; *AhAPX109, AhAPX111*, and *AhAPX13* expressed in stem, roots and peg; *AhAPX135* expressed in pericarp; and *AhAPX138* expressed in cotyledon (Figure 11). Particularly, a few genes also exhibited modest expressions in a variety of tissues. On the whole, expression dataset shows that some particular genes may substantially participate in peanut growth and development. Hence, the functional characterization of these genes may perhaps be carried out in future studies.

### Expression Profiling of *AhAPX* Genes Under Abiotic Stress and Hormones Treatments

**FIGURE 12:**
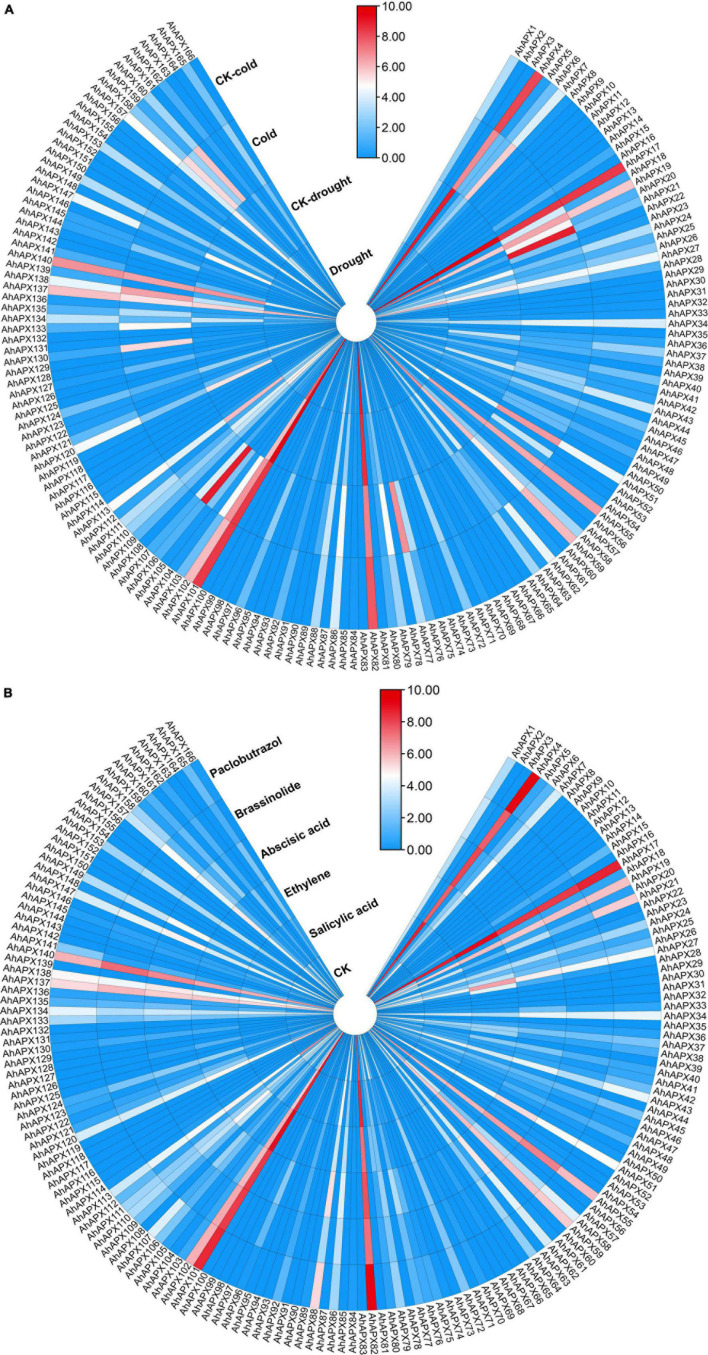
Expression profiling of *AhAPX* genes under different **(A)** abiotic stress, including cold and drought stress, and **(B)** phytohormone treatments such as abscisic acid, salicylic acid, ethylene, brassinolide, and paclobutrazol. In both maps, the label “CK” refers to the control plants in comparison to the stress-treated plants. In the expression bar, the red, white and blue colors show high to low expression levels. The circular heat map was designed by taking log2 of fragments per kilobase of transcript per million (FPKM) values.

To further study the contribution of all *AhAPX* genes toward abiotic and hormones stress tolerance in peanut, an openly available transcriptome dataset was used to evaluate the expression levels (Figure 12 and Supplementary Table 9). Similar to tissue-specific trend, only a few genes showed higher expressions in both cold and drought stresses. For instance, *AhAPX4, AhAPX17, AhAPX19, AhAPX82, AhAPX101*, and *AhAPX102* were highly expressed under stress (cold and drought) and CK conditions. Likewise, some genes also showed moderate expression levels, such as *AhAPX27, AhAPX34, AhAPX51, AhAPX55, AhAPX59, AhAPX113, AhAPX137, AhAPX138, AhAPX140*, and *AhAPX157* under stress (cold and drought) and normal conditions. On the other hand, *AhAPX720, AhAPX21, AhAPX51, AhAPX77, AhAPX106, AhAPX130, AhAPX158*, and *AhAPX160* displayed considerable expression under cold stress compared to CK conditions (Figure 12A).

Under phytohormones treatments, *AhAPX4, AhAPX17, AhAPX55, AhAPX59, AhAPX82, AhAPX101, AhAPX102, AhAPX137*, and *AhAPX140* displayed significantly higher expression patterns throughout the treatments. In comparison to CK, some genes are specifically expressed under certain hormones, such as *AhAPX21* under paclobutrazol, *AhAPX27* under ethylene and abscisic acid, *AhAPX51* under abscisic acid, and *AhAPX88* under abscisic acid and paclobutrazol (Figure 12B). Notably, most of the genes did not show any expression under any type of stress conditions. The candidate genes with higher expression could be genetically engineered to improve the tolerance against multiple hormones and abiotic stress (cold and drought) conditions.

### qRT-PCR-Based Expression Profiling of *AhAPX* Genes Under Cold and ABA Treatment

**FIGURE 13:**
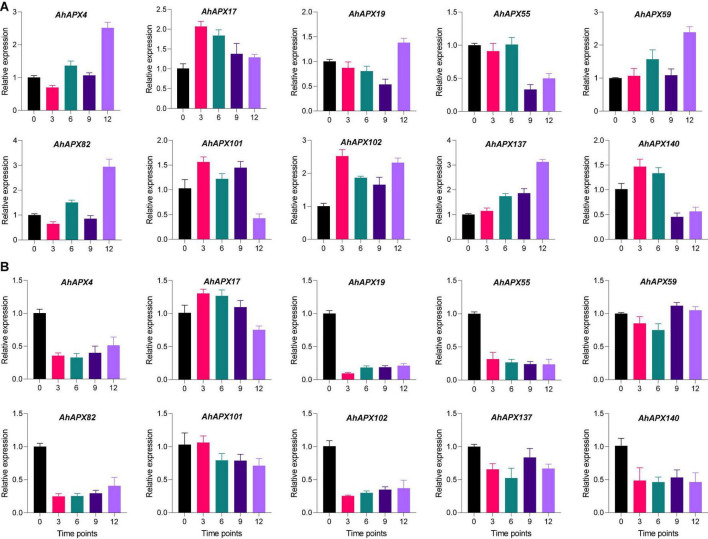
Expression profiling of the *AhAPX* genes under **(A)** abscisic acid and **(B)** cold stress treatments. The time points include 0 (CK), 3, 6, 9, and 12 h when the samples were picked for expression analysis after the stress treatment. Error bars signify the SD of three biological repeats (*n* = 3).

For qRT-PCR-based expression profiling, 10 highly upregulated *AhAPX* genes were selected based on transcriptome datasets to validate their transcript levels under ABA and cold treatment at various time points (Figure 13). Under ABA treatment, almost all genes demonstrated higher expression levels at all time points compared to CK, excluding a few cases. Such as, *AhAPX55* and *AhAPX140* showed relatively low expression at 9 and 12 h compared to CK and other time points (Figure 13A). In response to cold stress, although all the genes were upregulated; nevertheless, some genes showed relatively low expression levels compared to CK, such as *AhAPX4, AhAPX19, AhAPX55, AhAPX82, AhAPX102, AhAPX137*, and *AhAPX140*. Whereas *AhAPX17* and *AhAPX59* showed considerably higher expression than CK (Figure 13B). In short, all the preferred genes display parallel expression trends (i.e., upregulated) to those developed from transcriptome datasets (Supplementary Figure 4), therefore representing the reliability of the transcriptome datasets.

## Discussion

### Characterization and Evolution of *APX* Gene Family in Plants

Cultivated peanut is widely known as an essential oilseed, protein-enrich food crop worldwide and retains important breeding traits during domestication (Zhuang et al., 2019; Bohra et al., 2022). Even so, peanut production is still substantially influenced by numerous biotic and abiotic factors (Agarwal et al., 2018; Gangurde et al., 2020, 2021; Kumar et al., 2020; Shasidhar et al., 2020; Sinha et al., 2020; Jadhav et al., 2021; Pandey et al., 2021; Soni et al., 2021; Aravind et al., 2022; Bomireddy et al., 2022; Liu et al., 2022; Patel et al., 2022). When plants are exposed to diverse abiotic and biotic factors, APX enzyme as a primary marker can quickly eliminate unnecessary H_2_O_2_ (i.e., ROS scavenging) from plant cells by adjusting several physiological and biochemical activities to safeguard cells from the noxiousness of overproduction of ROS (Das and Roychoudhury, 2014; Mittler, 2017; Hasanuzzaman et al., 2020, 2021). During the past few years, excessive advancement has been achieved in studying the mode of action of *APX* genes; however, their vital role still requires more examination. Recent peanut genome sequencing data allow us to comprehensively discover new gene family members and recognize their functional and defensive mechanisms against stress conditions.

Usually, *APX* gene family of plants comprises a few genes. In this study, 166 *AhAPX* genes have been discovered in peanut genome (Supplementary Table 10), a larger *APX* family than previously reported *APX* gene families in watermelon (Malambane et al., 2018), shrub (Wang et al., 2022), rice (Teixeira et al., 2004), *A. thaliana* (Panchuk et al., 2002, Panchuk et al., 2005), sorghum (Akbudak et al., 2018), kiwifruit (Liao et al., 2020), tomato (Najami et al., 2008), wheat (Tyagi et al., 2020), and cotton (Tao et al., 2018). Deviations in the *APX* members amongst diverse plant species may perhaps be attributed to gene duplication events involving tandem and segmental repeats and play a role in expanding *APXs* for deviation. Repetition of *APX* genes was also discovered in several plant species (Teixeira et al., 2004; Panchuk et al., 2005; Akbudak et al., 2018; Liao et al., 2020; Wang et al., 2022). Our outcomes confirmed that *AhAPXs* had suffered segmental duplications (Supplementary Table 3). Subsequently, these reports recommended that *AhAPXs* duplicate cases may possibly play an essential role in gene evolution.

Previous studies showed that *APX* family genes are usually clustered into four major groups based on their subcellular localization or tree topologies (Pandey et al., 2011; Malambane et al., 2018; Tyagi et al., 2020). In the present study, all *APX* genes from four plant species were grouped into 11 main groups based on tree topologies and sequence similarities (Figure 2). This grouping was also recently supported by a new study on brassica crops (*B. napus* and *B. rapa*), where all *APX* genes were grouped into 13 subfamilies (Ma et al., 2021). Further, gene structure analysis also showed that genes belonging to the same subtree possess almost similar exon-intron patterns, ranging from 16 to 1 (exons) and 0 to 15 (introns) (Figure 5B and Supplementary Table 10). A significant difference was observed in gene structures where some genes have many exons-introns while some lack introns. Similar gene structure patterns were also reported in previous reports, such as in wheat number of exons extended from 7 to 12 (Tyagi et al., 2020). In *Actinidia chinensis*, the gene structure possesses 4–22 introns (Liao et al., 2020), which are higher than our observations. The exon-intron organization disparity was practiced by three important methods (exon/intron gain/loss, exonization/pseudoexonization, and insertion/deletion), and they are clearly supported by structural inconsistency (Xu et al., 2012). Notably, the *AhAPX* genes in each group almost exhibited comparable exon-intron group and conserved motifs (Figure 5), suggesting that these genes may possibly contribute to the similar tasks associated with several abiotic stressors. These outcomes are in agreement with earlier studies of kiwifruit (Liao et al., 2020), and wheat (Tyagi et al., 2020), where genes inside the same group comprise distinct structures and motifs organizations.

### The Contribution of *APX* Genes Toward Stress Responses and Tolerance Mechanisms

To boost our understanding into the involvement of *AhAPX* genes contrary to numerous environmental factors, *cis*-elements were predicted in the promoter of *AhAPX* genes. The discoveries showed that three types of *cis*-elements were recognized, i.e., abiotic stress, phytohormones, and growth and development-related elements (Figures 6, 7). Recent studies show that the *cis*-elements in *APX* genes contribute to the plant abiotic and phytohormones stress responses. Similar types of abiotic and phytohormone-related *cis*-regulatory elements have been identified in previous studies (Akbudak et al., 2018; Malambane et al., 2018; Tao et al., 2018; Liao et al., 2020; Tyagi et al., 2020; Wang et al., 2022). Furthermore, *AhAPXs* gene functions were further predicted by GO enrichment analysis (Figure 10), which also supported the role of these genes in ROS scavenging and stress response mechanisms. To get further insights into the role of *AhAPX* genes, their expression levels were examined under various hormones and abiotic stress treatments (Figures 12, 13). Our results showed that a few genes significantly contribute to specific stress responses like cold, drought, and ABA. These results are in agreement with the previous reports of Akbudak et al. (2018), where some *SbAPX* genes were significantly induced by drought stress in the leaves and roots of two genotypes. Similarly, many genes showed higher expression levels in *A. nanus* under cold and osmotic stress (Wang et al., 2022). Many *BrAPX* and *BnAPX* genes showed higher expression trends in cold-tolerant varieties in response to cold stress (Ma et al., 2021). Under drought stress, most of the *ClAPX* genes were significantly upregulated and displayed elevated expression in watermelon (Malambane et al., 2018). These conclusions can enhance our perception of *AhAPX* genes under various stress conditions, especially cold and drought.

Recent reports also suggest that manipulating *APX* genes could contribute to stress tolerance in plants. For instance, a novel *ScAPX6* gene from sugarcane was overexpressed in tobacco (*Nicotiana benthamiana*), and transgenic plants showed improved resistance to the biotic stress (*Pseudomonas solanacearum* and *Fusarium solani*) by positively regulating the phytohormones contents (Liu et al., 2018). The overexpression of *PcAPX* from Chinese white poplar (*Populus tomentosa*) improves tolerance to multiple stresses, including salinity, drought, and oxidative stress in transgenic tobacco plants by improving biochemical mechanisms (Cao et al., 2017). Likewise, the overexpression of Populus peroxisomal *PpAPX* gene enhances drought stress tolerance in transgenic tobacco plants (Li et al., 2009). Transgenic tobacco overexpressing cytosolic *APX* gene alleviated the drought stress tolerance (Faize et al., 2011). Ectopic overexpression of the peroxisomal *SbpAPX* gene improves salinity tolerance in transgenic peanut (Singh et al., 2014). So far, this is the only *APX* gene that has been functionally characterized in peanut. These studies recommend that the genetic engineering of *APX* genes is of great importance in conferring various stress tolerance in crop plants, including peanut.

Among various identified TFs, ERF TFs have been functionally characterized from peanut. The results exhibited that overexpression of *AhERF008* impaired the root magnitude of *A. thaliana*; however, overexpression of *AhERF019* improved tolerance to heat, salinity and drought stresses in *A. thaliana* (Wan et al., 2014). Ectopic overexpression of MYB repressor gene (*GmMYB3a*) increases drought tolerance and physiological mechanisms in transgenic peanut under drought stress (He et al., 2020). Another NAC TF gene (*AhANC4*) from peanut enhances drought tolerance in transgenic tobacco plants due to improved stomatal closure and advanced water use efficiency (Tang et al., 2017). A novel WRKY TF gene (*AhWRKY75*) improved salinity tolerance in transgenic peanut plants by improving antioxidant mechanisms, ROS scavenging, stomatal conductance, and photosynthesis under salinity stress (Zhu et al., 2021). All these studies suggest that the genetic engineering of TF is a promising approach to improve peanut performance under stressed conditions.

### The Contribution of *APX* Genes in Numerous Organs/Tissues

Here, the tissue-specific expression profiling of 166 *AhAPX* genes was carried out in various organs/tissues using publically available transcriptome datasets. Overall, the results showed that only a few *AhAPX* genes showed higher expression levels, particularly in roots, stem, leaf, peg, pericarp, testa, and flowers (Figure 11). In *A. nanus*, the RNA-seq data was used to observe the expression levels in leaves. The results displayed that only one gene showed substantially higher expression in leaf (Wang et al., 2022). In wheat, most of the genes showed higher expression patterns in root, stem, leaf, spike, and grain. Especially, almost half of the *APX* genes were found to be leaf-specific due to significantly higher expression (Tyagi et al., 2020). In *A. chinensis*, qRT-PCR-based expression profiling of 13 *AhAPX* genes was performed in various fruit developmental stages. The outcomes demonstrated that eight *AcAPX* genes had the utmost expression patterns during the color turn-off phase (Liao et al., 2020). It can be concluded that the tissue-specific *APX* genes (such as *AhAPX4, AhAPX17, AhAPX77, AhAPX82, AhAPX101*, and *AhAPX130*) could be considered as target candidates for further molecular studies to fully reveal their role and mechanisms in peanut growth and development.

### MicroRNA: Emerging Players for Crop Improvement and Stress Tolerance

MicroRNAs (miRNAs) are a group of tiny-non-coding RNAs formed from individual-strand hairpin RNA precursors. These miRNAs switch gene expression by attaching to corresponding sequences surrounded by target mRNAs (Jamla et al., 2021; Patil et al., 2021). Extensive progress has been put together in finding the targets of peanut miRNAs that contribute to various stresses and developmental activities (Zhao et al., 2010, 2015; Chi et al., 2011; Zhang et al., 2017; Chen H. et al., 2019; Figueredo et al., 2020; Tong et al., 2021). The present predicted 14 miRNAs belonging to 12 different families targeting 33 *AhAPX* genes (Figure 8 and Supplementary Table 6). Notably, none of the previous studies predicted the miRNAs that can target *APX* genes, expect one study. A recent study supports our findings where 51 miRNAs have been identified targeting 29 *TaAPX* genes in wheat (Tyagi et al., 2020). However, these target genes are yet to be characterized in wheat. In another study, a new miRNA (ath-miR447a-3p) was found to be targeting *APX3* gene, and its expression analysis showed that it negatively regulated the expression of *APX3*, which is directly involved in the APX synthesis under drought stress in *Zanthoxylum bungeanum* (Fei et al., 2020).

However, some of the identified miRNAs have been reported to take part in stress tolerance and developmental processes. For instance, spatio-temporal expression patterns of miRNA159 family representatives have been found targeting *MYB* genes in grapevine (*Vitis vinifera* L.). The results showed that *miRNA159c-VvGAMYB* module is involved in gibberellin-tempted parthenocarpy in grapevine (Wang et al., 2018). Another study discovered that miR167A is the main member of miR167 family that regulates the *A. thaliana* reproduction. Further, miR167A acts as a parental gene that works mostly *via ARF6* and *ARF8* genes in maternal management of embryonic and seed growth (Yao et al., 2019). A member of miRNA156 family has been reported to be involved in the interaction between ABA and miRNA156, which regulates the expression profile of anthocyanin biogenesis genes in drought-stressed plants (González-Villagra et al., 2017). Notably, several miRNA families such as miR3513, miR3518, miR3520, miR3513, miR3516, etc., have not been functionally characterized; therefore, the future work could also be focused on these unique miRNAs to reveal their potential in plant growth and development. Moreover, the expression profiling of prophesied miRNAs and their targeted genes demands validation to direct their biological roles in the peanut breeding programs.

## Conclusion

Altogether, we recognized 166 putative *AhAPX* genes in the cultivated peanut genome, which are mapped on all chromosomes, including unassembled ones. Comprehensive *in silico* examination of *AhAPX* genes, i.e., characterization, evolution, gene structure, conserved motifs, *cis*-elements, putative miRNA and TFs prediction, GO and KEGG enrichment were executed to increase our understanding of *AhAPX* genes in peanut. Their expression trends were also evaluated in various developmental organs/tissues, phytohormones, and abiotic stress conditions. In brief, this report set the foundation for further functional experiments (such as overexpression, gene editing *via* CRISPR/Cas system, etc.) of some candidate genes such as *AhAPX4/17/19/55/59/82/101/102/137* and *AhAPX140*, which can advance the peanut breeding programs under undesirable stress conditions.

## Data Availability Statement

The datasets presented in this study can be found in online repositories. The names of the repository/repositories and accession number(s) can be found below: NCBI BioProject–PRJNA480120.

## Author Contributions

AR and WZ conceived the idea. AR analyzed the data and wrote the manuscript. YS and KC helped with qRT-PCR analysis. YS, KC, CZ, LW, HF, AC, and HC helped in literature search and data analysis. WZ and RKV supervised the research, and reviewed and improved the manuscript. All authors have read and approved the final version of the manuscript.

## Conflict of Interest

The authors declare that the research was conducted in the absence of any commercial or financial relationships that could be construed as a potential conflict of interest.

## Publisher’s Note

All claims expressed in this article are solely those of the authors and do not necessarily represent those of their affiliated organizations, or those of the publisher, the editors and the reviewers. Any product that may be evaluated in this article, or claim that may be made by its manufacturer, is not guaranteed or endorsed by the publisher.

## Footnotes

1
http://peanutgr.fafu.edu.cn/

2
http://www.arabidopsis.org/

3
http://www.hmmer.org/

4
http://pfam.xfam.org/

5
https://www.peanutbase.org/

6
http://web.expasy.org/protparam/

7
http://cello.life.nctu.edu.tw/

8
https://github.com/CJ-Chen/TBtools

9
https://meme-suite.org/meme/db/motifs

10
https://megasoftware.net/home

11
https://itol.embl.de/

12
http://bioinformatics.psb.ugent.be/webtools/plantcare/html/

13
https://www.zhaolab.org/psRNATarget/home

14
https://cytoscape.org/download.html

15
http://eggnog-mapper.embl.de/

16
http://plantregmap.gao-lab.org/binding_site_prediction.php

17
https://www.graphpad.com/
