# Supplementary material for: Genome-Wide Characterization of Ascorbate Peroxidase Gene Family in Peanut (Arachis hypogea L.) Revealed Their Crucial Role in Growth and Multiple Stress Tolerance
Source: Front Plant Sci. 2022 Sep 9;13:962182. doi: 10.3389/fpls.2022.962182 (PMC9524023; doi:10.3389/fpls.2022.962182)
Supplement: Supplementary file 1 [file Data_Sheet_1.ZIP › Supplementary Figures.docx]

**Supplementary Figures**


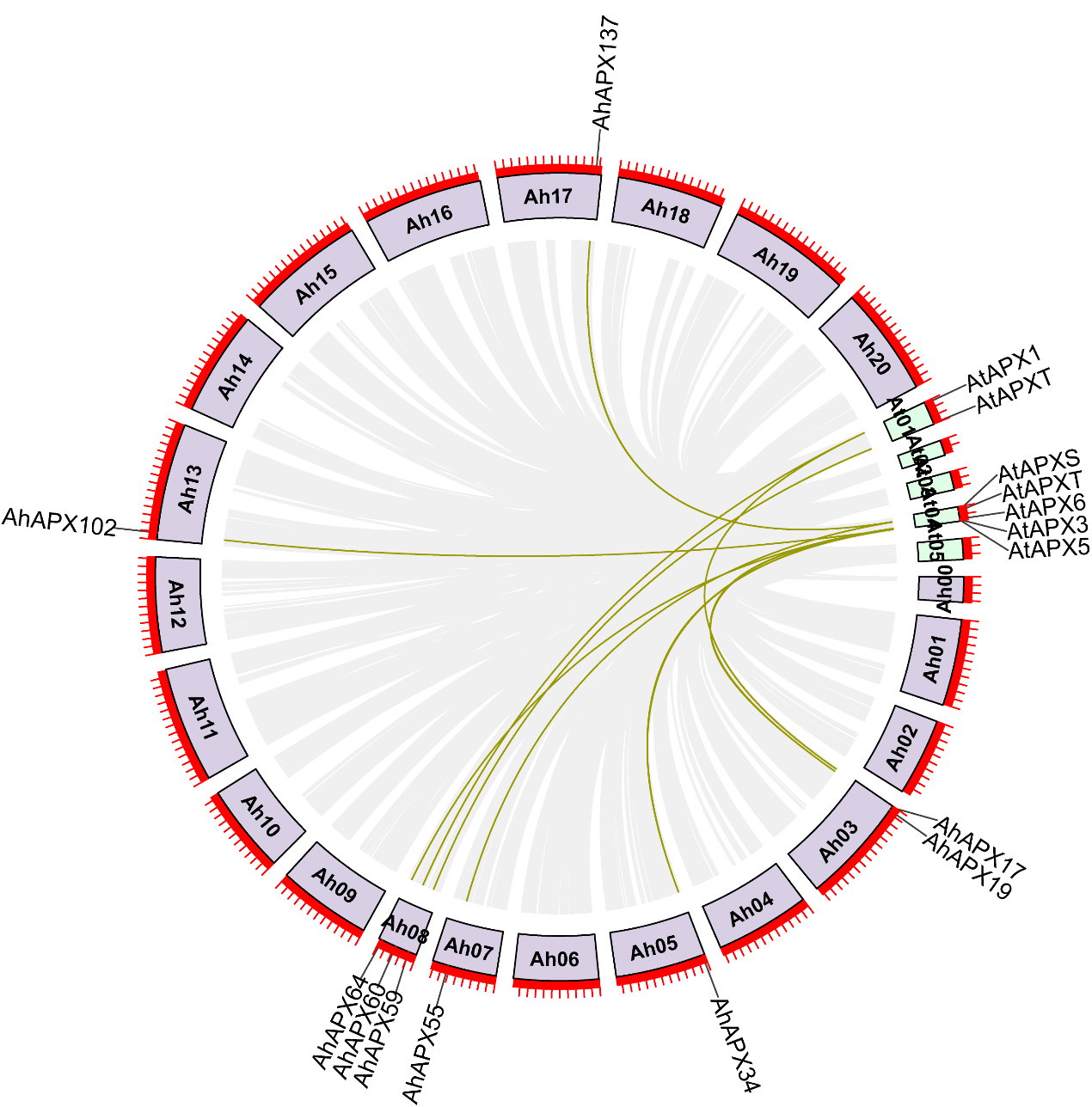
**Supplementary Figure 1.** Chromosomal positions and inter-chromosomal groups of duplicated gene pairs between *A. thaliana* and *A. hypogea*. Grey lines in the background demonstrate all syntenic blocks in the *A. hypogea* genome, and the brownish lines exhibit the segmental or tandem duplication network zones among *APX* genes from both genomes. The location of *APX* genes is marked with black lines outside with chromosome names.


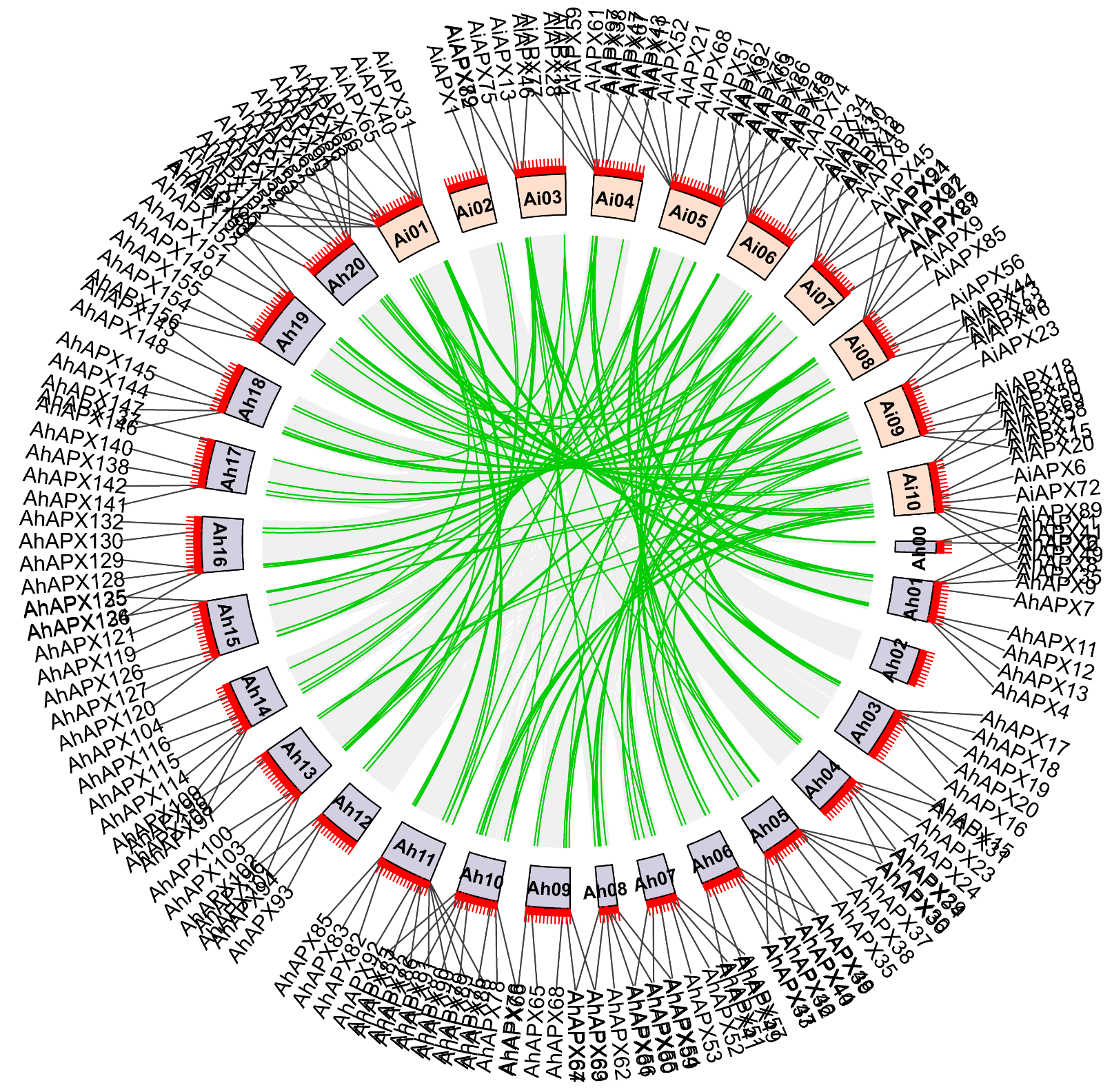
**Supplementary Figure 2.** Chromosomal positions and inter-chromosomal groups of duplicated gene pairs between *A. hypogea* and *A. ipaensis.* Grey lines in the background demonstrate all syntenic blocks in the *A. hypogea* genome, and the green lines exhibit the segmental or tandem duplication network zones among *APX* genes from both genomes. The location of *APX* genes is marked with black lines outside with chromosome names.


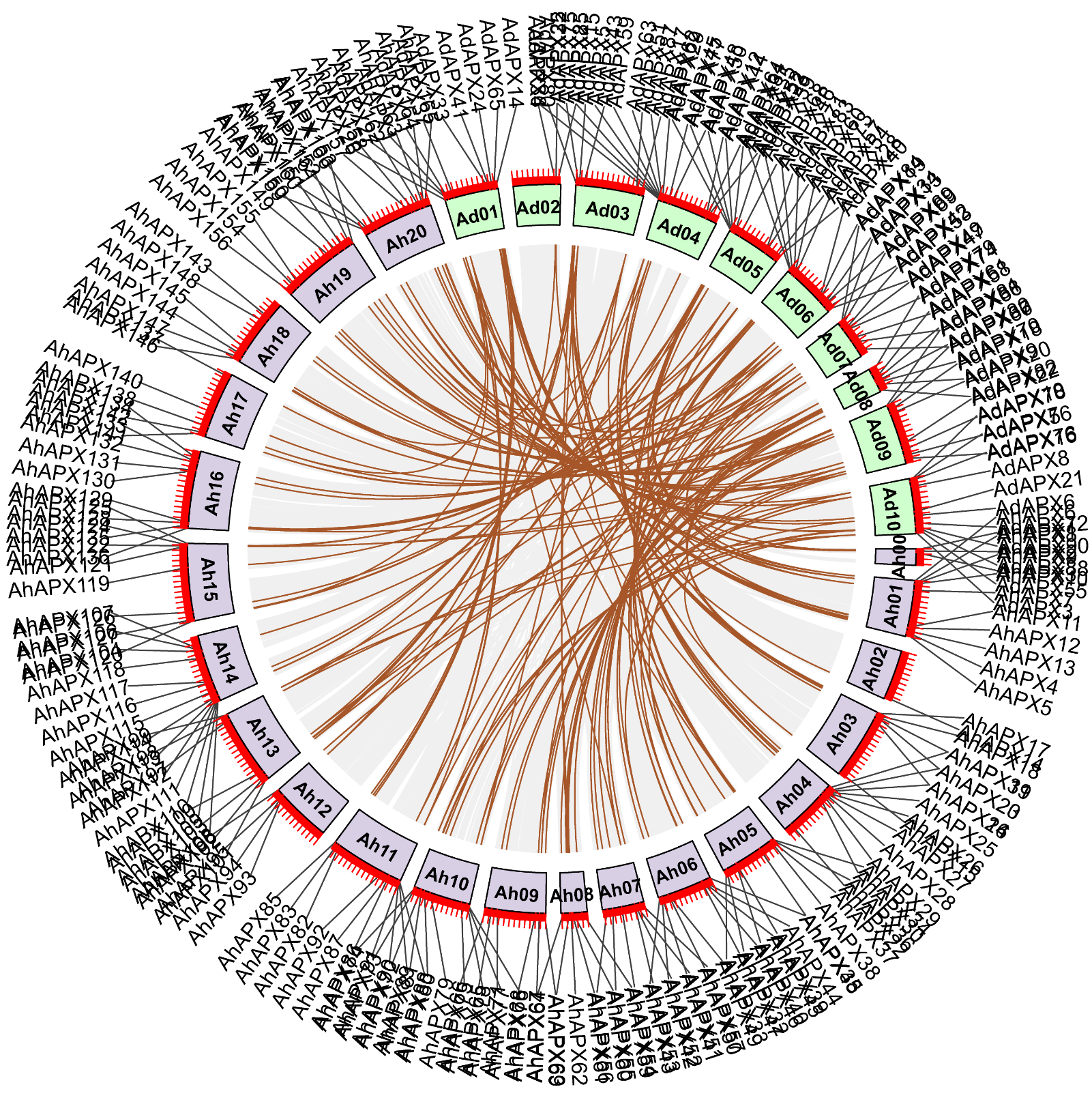
**Supplementary Figure 3.** Chromosomal positions and inter-chromosomal groups of duplicated gene pairs between *A. duranesis* and *A. hypogea*. Grey lines in the background demonstrate all syntenic blocks in the *A. hypogea* genome, and the dark brown lines exhibit the segmental or tandem duplication network zones among *APX* genes from both genomes. The location of *APX* genes is marked with black lines outside with chromosome names.

**
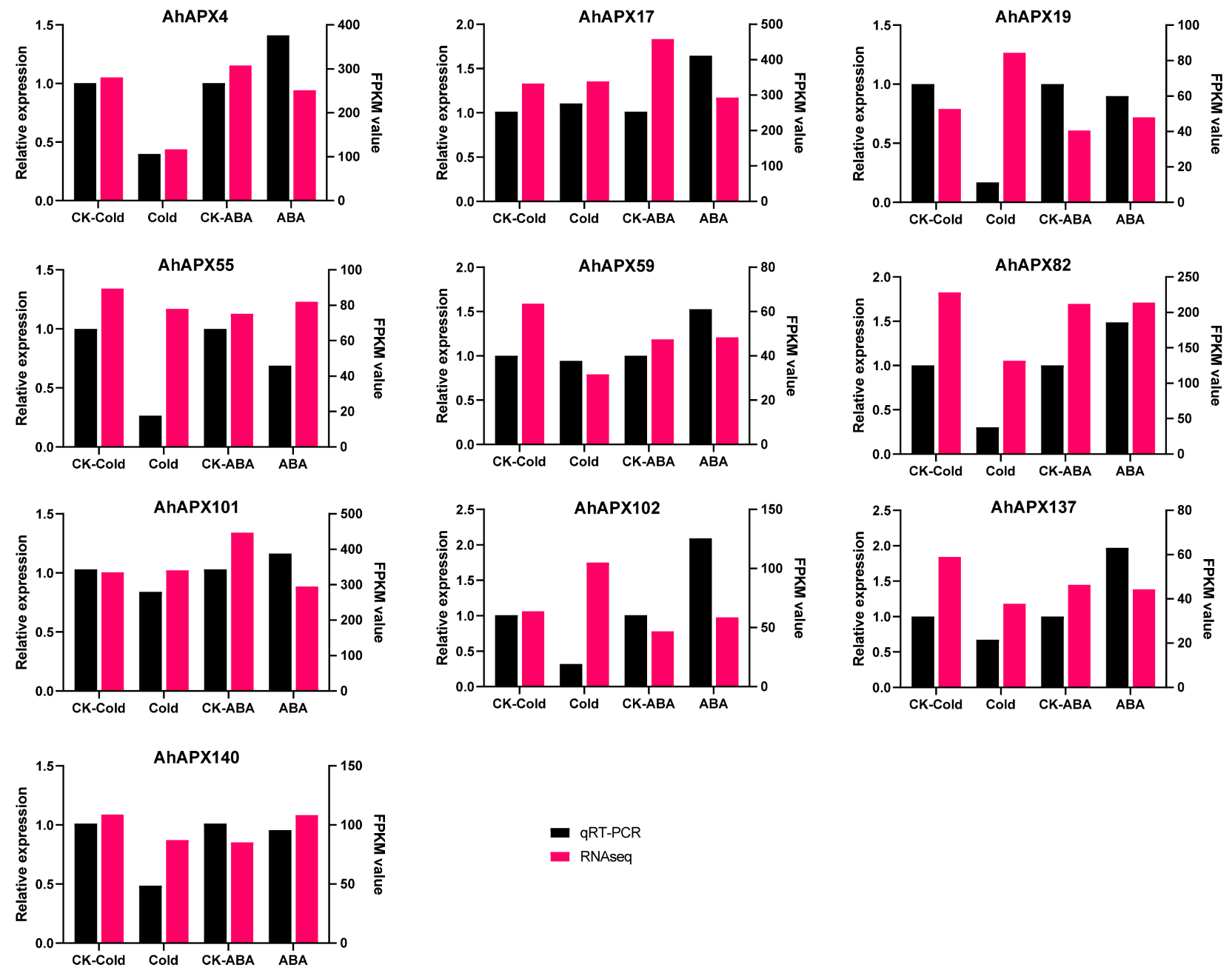
Supplementary Figure 4.** Comparisons of expression profiling of *AhAPX* genes under cold and ABA treatments. The left side indicates the relative expression levels of qRT-PCR results, while the right side indicates the FPKM values of RNAseq datasets. The graphs were constructed using the mean values of different time points to verify the transcriptome datasets.
